# Eicosapentaenoic and docosahexaenoic acids attenuate hyperglycemia through the microbiome-gut-organs axis in *db/db* mice

**DOI:** 10.1186/s40168-021-01126-6

**Published:** 2021-09-10

**Authors:** Pan Zhuang, Haoyu Li, Wei Jia, Qiyang Shou, Ya’er Zhu, Lei Mao, Wenqiao Wang, Fei Wu, Xiaoqian Chen, Xuzhi Wan, Yuqi Wu, Xiaohui Liu, Yin Li, Fanghuan Zhu, Lilin He, Jingnan Chen, Yu Zhang, Jingjing Jiao

**Affiliations:** 1 National Engineering Laboratory of Intelligent Food Technology and Equipment, Zhejiang Key Laboratory for Agro-Food Processing, College of Biosystems Engineering and Food Science, Zhejiang University, Hangzhou, 310058 Zhejiang China; 2 The Second Clinical Medical College, Zhejiang Chinese Medical University, Hangzhou, 310005 Zhejiang China; 3 Analysis Center of Agrobiology and Environmental Sciences, Zhejiang University, Hangzhou, 310058 Zhejiang China; 4 Department of Nutrition, School of Public Health, Department of Clinical Nutrition of Affiliated Second Hospital, Zhejiang University School of Medicine, Hangzhou, 310058 Zhejiang China

**Keywords:** Eicosapentaenoic acid, Docosahexaenoic acid, Gut microbiome, Gut metabolome, Diabetes

## Abstract

**Background:**

Eicosapentaenoic acid (EPA) and docosahexaenoic acid (DHA) have been suggested to prevent the development of metabolic disorders. However, their individual role in treating hyperglycemia and the mechanism of action regarding gut microbiome and metabolome in the context of diabetes remain unclear.

**Results:**

Supplementation of DHA and EPA attenuated hyperglycemia and insulin resistance without changing body weight in *db/db* mice while the ameliorative effect appeared to be more pronounced for EPA. DHA/EPA supplementation reduced the abundance of the lipopolysaccharide-containing Enterobacteriaceae whereas elevated the family Coriobacteriaceae negatively correlated with glutamate level, genera *Barnesiella* and *Clostridium XlVa* associated with bile acids production, beneficial *Bifidobacterium* and *Lactobacillus*, and SCFA-producing species. The gut microbiome alterations co-occurred with the shifts in the metabolome, including glutamate, bile acids, propionic/butyric acid, and lipopolysaccharide, which subsequently relieved β cell apoptosis, suppressed hepatic gluconeogenesis, and promoted GLP-1 secretion, white adipose beiging, and insulin signaling. All these changes appeared to be more evident for EPA. Furthermore, transplantation with DHA/EPA-mediated gut microbiota mimicked the ameliorative effect of DHA/EPA on glucose homeostasis in *db/db* mice, together with similar changes in gut metabolites. In vitro, DHA/EPA treatment directly inhibited the growth of *Escherichia coli* (Family Enterobacteriaceae) while promoted *Coriobacterium glomerans* (Family Coriobacteriaceae), demonstrating a causal effect of DHA/EPA on featured gut microbiota.

**Conclusions:**

DHA and EPA dramatically attenuated hyperglycemia and insulin resistance in *db/db* mice, which was mediated by alterations in gut microbiome and metabolites linking gut to adipose, liver and pancreas. These findings shed light into the gut-organs axis as a promising target for restoring glucose homeostasis and also suggest a better therapeutic effect of EPA for treating diabetes.

Video abstract

**Supplementary Information:**

The online version contains supplementary material available at 10.1186/s40168-021-01126-6.

## Background

Type 2 diabetes (T2D), an obesity-related metabolic disease, is rising exponentially throughout the world [1]. Genetic susceptibility and lifestyle factors are recognized as the main cause of T2D. Intriguingly, recent evidence suggests non-communicable chronic diseases, such as diabetes, could be communicable via dysbiotic microbiota, as demonstrated by fecal microbiota transplant (FMT) studies [2–4]. Previous literature has also revealed a critical role of dietary factors, including dietary fat [5], in shaping gut microbial composition. Increased consumption of saturated fat induces intestinal dysbacteriosis and endotoxemia [6, 7], whereas polyunsaturated fatty acids (PUFAs) restore gut micro-ecological equilibrium and relieve inflammation [8–11]. Notably, emerging evidence shows that marine n-3 PUFAs, eicosapentaenoic acid (EPA) and docosahexaenoic acid (DHA), are able to ameliorate insulin resistance (IR) in rodents probably via regulating adipocytokines secretion [12–14], inhibiting adipose remodeling [15], lowering inflammation [16–18], and enhancing mitochondrial function and β-oxidation [19]. Although fish oil, rich in n-3 PUFAs, was reported to alter intestinal microbiota composition [10, 20, 21], another study showed that n-3 PUFAs did not protect against gut microbiota dysbiosis in mice [22]. Importantly, data regarding the individual effect of DHA versus EPA on the gut microbiome in the context of diabetes is absent.

Emerging evidence suggests a complex interplay between gut and host organs [23–26], including adipose, liver, and pancreas. Gut dysbiosis increases the influx of lipopolysaccharide (LPS), which further triggers systemic inflammation and IR by activating toll-like receptor 4 (TLR4) [27]. In addition, the microbiota-derived metabolites short-chain fatty acids (SCFAs), including propionate and butyrate, promote the glucagon-like peptide-1 (GLP-1) production from intestinal L cells and thereby improve the release of insulin from the pancreas [28, 29]. SCFAs are also signaling molecules that benefit metabolic health via activating G-protein coupled receptors (GPRs) [30]. Intestinal microbiota was also found to be capable of regulating enterohepatic bile acids (BAs) metabolism by biotransforming BAs into forms that possess potent regulatory effects on BA signaling receptors [31, 32]. Glutamate-fermenting commensal contributes to the improved metabolic health in obesity, probably by damping glutamate-induced IR [33]. Therefore, gut microbiota manipulated by dietary factors may, in turn, impact the host metabolism via microbiota-derived metabolites. However, how n-3 PUFAs affect gut metabolome remains poorly understood.

In the present study, we aimed to investigate the individual effects of DHA and EPA supplementation on glucose metabolism and possible crosstalk between gut and adipose, liver, and pancreas in the context of diabetes. To examine this hypothesis, we assessed the changes in the gut microbiome and metabolome in response to DHA/EPA supplementation in *db/db* mice and FMT was further applied to determine whether the beneficial effect was driven by gut microbiota and metabolites linking gut to adipose, liver, and pancreas. Finally, featured gut microbial species were treated with DHA/EPA in vitro to confirm whether DHA/EPA could directly affect their growth.

## Results

### EPA and DHA attenuate hyperglycemia and enhance energy expenditure in db/db mice

**Fig. 1:**
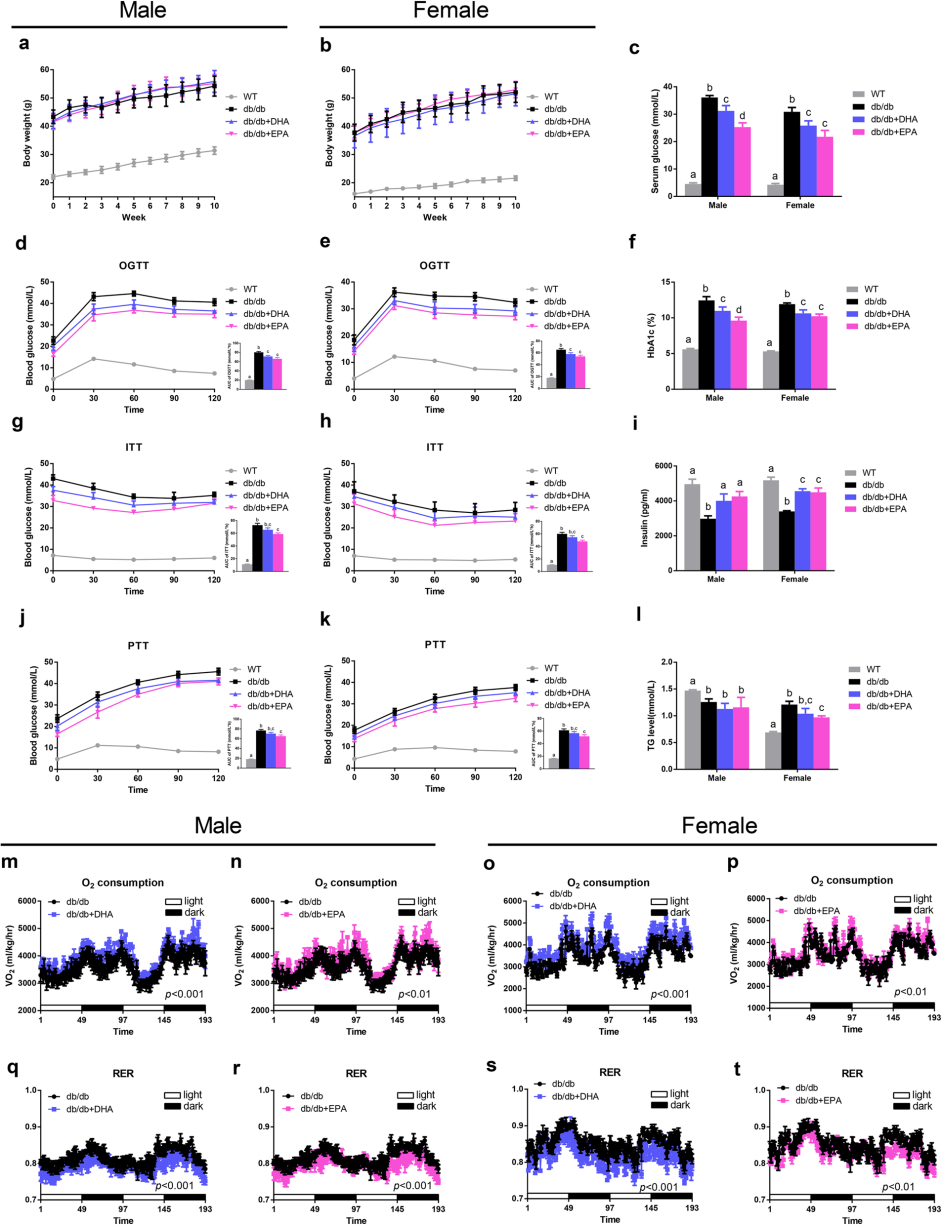
Differential effects of DHA and EPA on glucose homeostasis and metabolic profile in *db/db* mice. The *db/db* mice were fed either a control diet (*db/db*), DHA-enriched diet (*db/db* + DHA), or EPA-enriched diet (*db/db* + EPA) for 10 weeks (*n* = 6 male and *n* = 6 female per group). **a, b** Body weight curves. **c** Serum glucose levels. **d**, **e, g, h, j, k** Time-dependent profiles of serum glucose levels in OGTT, ITT, and PTT with AUC. **f** Blood HbA1c levels. **i** Serum insulin levels. **l** Serum TG levels. **m–p** Oxygen consumption. **q–t** RER. Data are presented as the mean ± SEM. Data with different superscript letters are significantly different (*P* < 0.05) using one-way ANOVA followed by Tukey’s multiple comparison post-test. *P* values in **m–t** were assessed by two-way ANOVA

To assess how DHA and EPA affect glucose homeostasis, we fed *db/db* mice isocaloric diets that were control diet (CD) supplemented with 1% (w/w) EPA or DHA for 10 weeks (Additional file 1: Table S1). Although food intake and weight gain were not affected during the intervention (Fig. 1a, b and Additional file 1: Figure S1a), both DHA and EPA supplemented mice exhibited significantly lower fasting glucose and HbA1c levels and a higher insulin level compared with *db/db* mice fed CD (Fig. 1c, f, i) after the treatment. Results from the oral glucose tolerance test (OGTT) and insulin tolerance test (ITT) demonstrated that both DHA and EPA improved glucose tolerance while EPA markedly increased insulin sensitivity (Fig. 1d, e, g, h). Additionally, EPA-fed mice had a higher pyruvate tolerance (Fig. 1j, k), which indicated inhibited gluconeogenesis. Together, treatment with DHA/EPA partially restored glucose homeostasis in *db/db*/ mice while EPA manifested a more potent ameliorative effect than DHA.

Regarding lipid metabolism, we observed EPA-fed female mice had significantly reduced levels of TG, TC, and LDL-C (Fig. 1l and Additional file 1: Figure S1d, e, and f). Although DHA/EPA supplementation did not change white adipose tissue (WAT) mass, interscapular brown adipose mass was enlarged by EPA and DHA (Additional file 1: Figure S1b, c). It is noteworthy that both DHA and EPA elevated oxygen consumption and decreased RER (Fig. 1m–t), indicative of enhanced energy expenditure and fat utilization.

### DHA and EPA differentially alter the gut microbiome

The effectiveness of the EPA/DHA intervention was verified by the dramatic increase in tissue levels of DHA and EPA (Additional file 1: Figure S2a). Meanwhile, we detected significant changes in antimicrobial peptide production in the small intestine: DHA feeding markedly increased the expression of α-defensin–Defa in male and female mice and Reg3g in females. The expressions of α-defensin–Defa, Pla2g2a, and Reg3g were upregulated 2.5–20-fold by EPA supplementation (Additional file 1: Figure S2b, c). Therefore, we performed 16S rRNA gene sequencing to analyze the differences in gut microbial ecology between groups.

Compared with WT mice, *db/db* mice had substantially lower bacterial richness whereas DHA and EPA feeding partially restored the phylogenetic diversity indicated by Chao1 (Additional file 1: Figure S3a–d). Principal coordinate analysis (PCoA) revealed distinct clustering of WT, *db/db*, and *db/db* + DHA/EPA groups (Additional file 1: Figure S3e, f), whereas EPA and DHA groups were not clearly separated. At the phylum level, DHA and EPA feeding significantly enriched Verrucomicrobia and Deferribacteres phyla in male mice while Bacteroidetes phylum was greater in DHA/EPA-fed female mice (Additional file 1: Figure S3g, h).

**Fig. 2:**
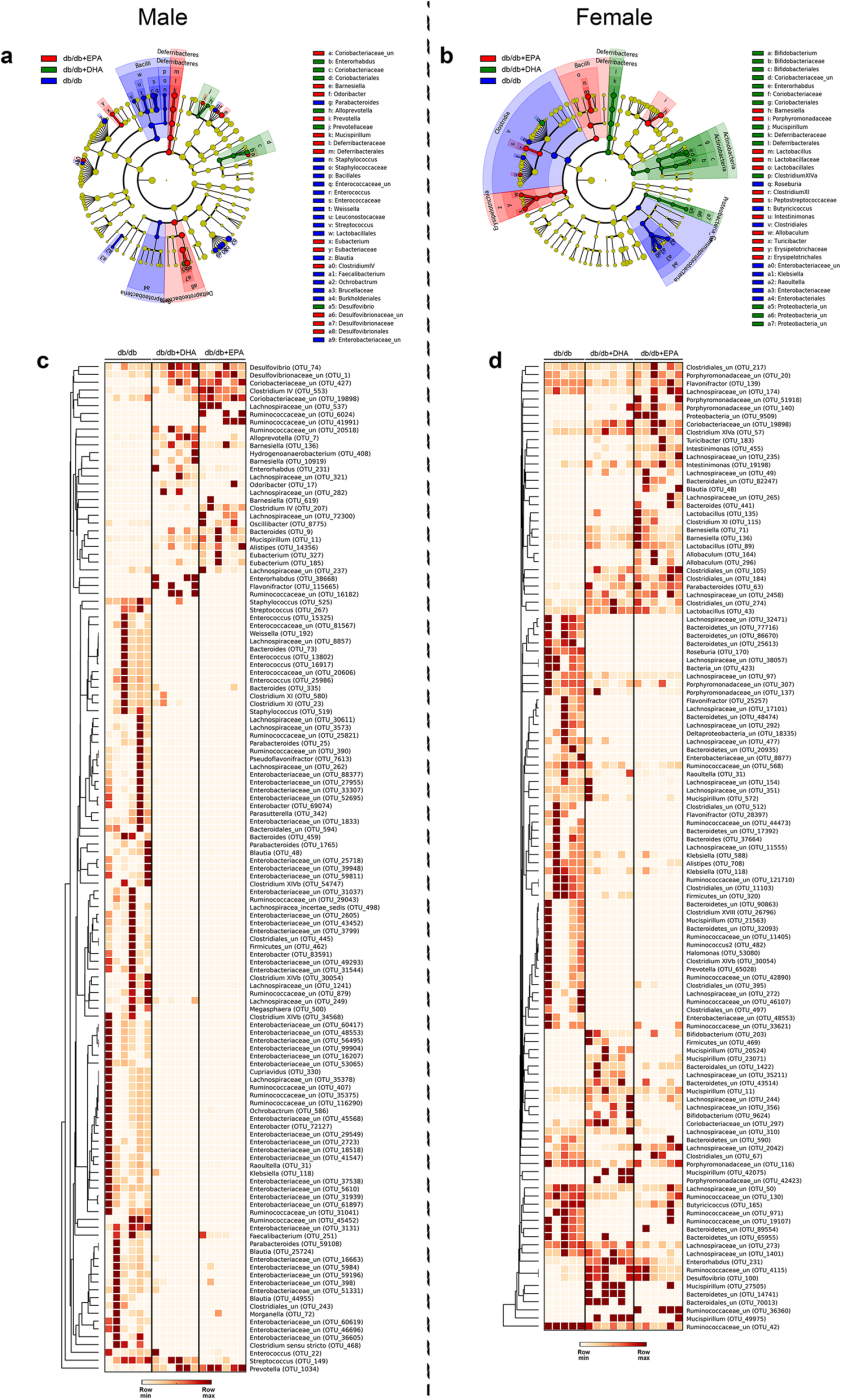
DHA and EPA differentially alter gut microbial phylotypes in *db/db* mice. Fecal microbiota composition in mice from different groups were analyzed using 16S rDNA sequencing (*n* = 6 male and *n* = 5-6 female per group). **a**, **b** Cladogram obtained from LEfSe analysis (LDA > 2), showing the most differentially abundant taxa enriched in microbiota from mice in *db/db* (blue), *db/db* + DHA (green), and *db/db* + EPA (red) groups. **c**, **d** Heatmap showing OTUs with significantly different relevant abundances between three treatment groups (as determined by Kruskall-Wallis test followed by Mann–Whitney test). Each row represents an OTU labeled by the lowest taxonomic description and OTU ID, normalized to the row maximum

We further performed linear discriminant analysis effect size (LEfSe) (LDA > 2) to identify taxa that may be microbiological markers for *db/db*, *db/db* + DHA, and *db/db* + EPA groups (Fig. 2a, b). In male mice, DHA and EPA supplementation significantly elevated the abundances of phylotypes Coriobacteriaceae, Desulfovibrionaceae, and *Prevotella* while reduced Enterobacteriaceae, Bacillales, Lactobacillales, *Staphylococcus*, *Streptococcus*, *Enterococcus*, *Parabacteroides*, *Weissella, Blautia*, and *Faecalibacterium*. Moreover, DHA specifically enriched *Alloprevotella* and *Enterorhabdus* while EPA specifically enriched Deferribacteraceae, *Barnesiella*, *Odoribacter*, *Mucispirillum*, *Clostridium lV*, and *Eubacterium*. In female mice, DHA and EPA feeding reduced the abundances of Enterobacteriaceae, Clostridiales, *Klebsiella*, *Raoultella*, and *Roseburia*. Additionally, DHA-fed female mice had more abundant Coriobacteriaceae, Deferribacteraceae, *Bifidobacterium, Enterorhabdus*, *Mucispirillum*, and *Clostridium XlVa*. EPA-fed female mice had greater species from *Barnesiella*, *Lactobacillus*, *Clostridium Xl*, *Intestinimonas*, Erysipelotrichaceae, *Allobaculum*, and *Turicibacter.* Heatmaps also reveal differential OTUs from the above phylotypes between three groups in males and females, respectively (Fig. 2c, d). Altogether, these findings demonstrated that DHA and EPA differentially modulated the dysbiosis of gut microbiota in *db/db* mice.

### EPA and DHA modulate gut metabolome

**Fig. 3:**
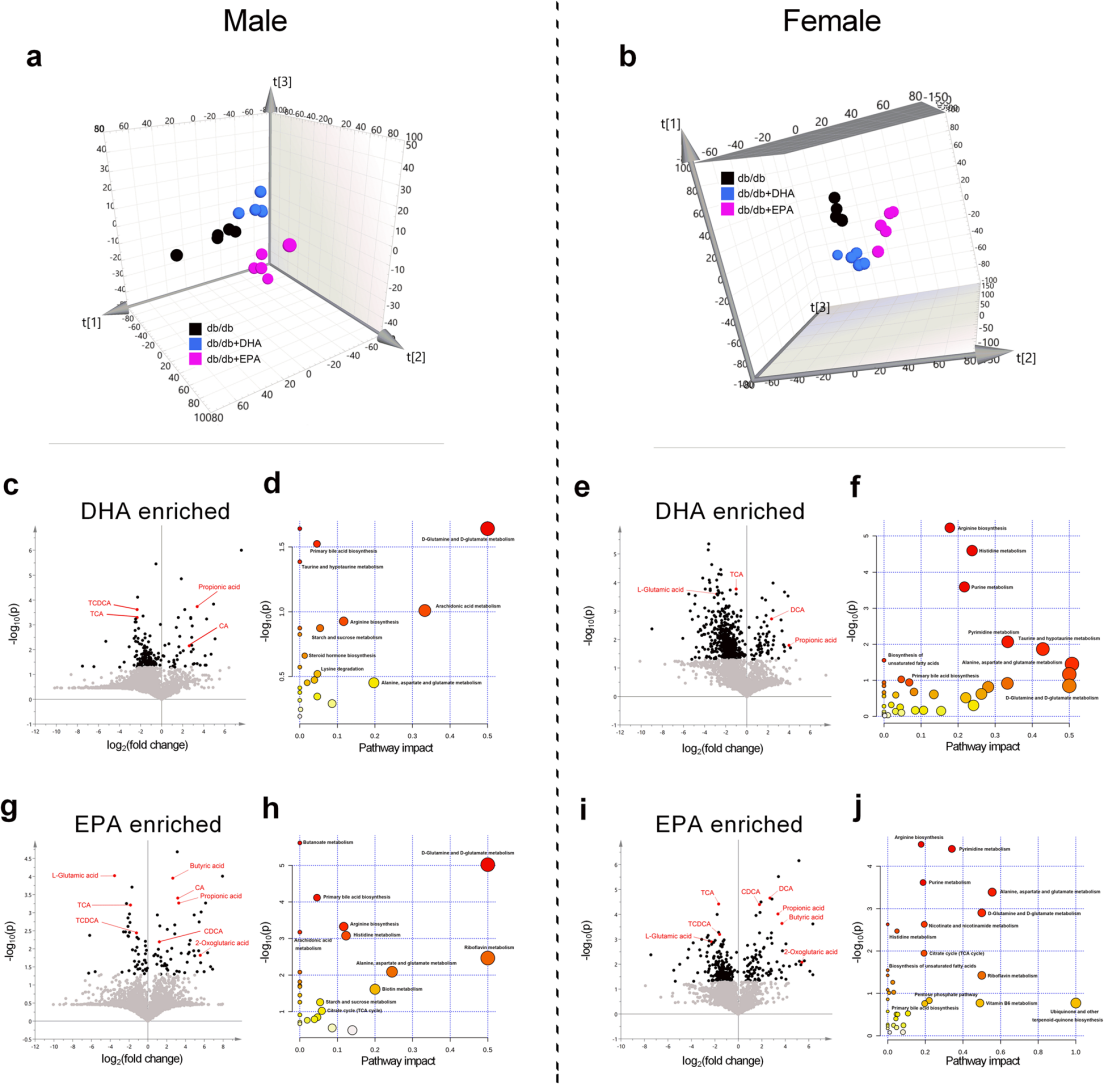
DHA and EPA differentially modulate gut metabolome in *db/db* mice. Global metabolic profiling on fecal was performed by UHPLC-Q-Orbitrap-HRMS (*n* = 5 male and *n* = 5 female per group). **a** Three-dimensional (3D) view of PCA score plot by UHPLC-Q-Orbitrap-HRMS analysis in male mice (combination of ions from positive and negative ion modes. *R*^2^*X* = 0.67, *Q*^2^ (cum) = 0.55). **b** 3D view of PCA score plot in female mice (*R*^2^*X* = 0.59, *Q*^2^ (cum) = 0.46). **c**,** e** Volcano plot (based on the combination of ions from positive and negative ionization modes) between *db/db* + DHA and *db/db* groups. **g, i** Volcano plot between *db/db* + EPA and *db/db* groups. Black points in the volcano plot represent significantly different metabolites which include key metabolites (highlighted in red) involved in glutamate metabolism and bile acid metabolism. **d, f** Pathway impact resulting from the differential metabolites (obtained from OPLS-DA model with VIP > 1) using MetaboAnalyst in DHA-fed mice. **h, j** Pathway impact resulting from the differential metabolites in EPA-fed mice. Small *P* value and big pathway impact factor indicate that the pathway is greatly influenced. Pathways of glutamate metabolism and bile acid metabolism were perturbed by DHA/EPA

Gut microbiota interacts with metabolites to impact the host metabolism [34]. We predicted changes in metabolic functions within the microbiome by Kyoto Encyclopedia of Genes and Genomes (KEGG) analysis of differentially expressed genes between DHA/EPA and *db/db* groups. Results showed that the pathways of ‘‘D-glutamine and D-glutamate metabolism’’ and ‘‘primary/secondary bile acid biosynthesis’’ were consistently upregulated by DHA/EPA treatment in male and female mice (Additional file 1: Figure S4). Therefore, we assessed the potential differences in fecal metabolite profiles between the three groups by an untargeted metabolomics approach. The partial least squares discriminant analysis (PLS-DA) showed a clear separation between the three groups according to the abundance of fecal metabolites (Fig. 3a, b). To maximize the discrimination between DHA/EPA-treated mice and CD-fed mice, we next performed pairwise OPLS-DA on the fecal metabolome data from these groups. As revealed by the volcano plots, substantial metabolic changes occurred after DHA/EPA supplementation in *db/db* mice (Fig. 3c, e, g, i). Among the significantly changed molecules (marked in black), we noted that l-glutamic acid, taurine-conjugated (T) cholic acid (CA), and chenodeoxycholic acid (CDCA) were markedly decreased while 2-oxoglutaric acid, CA, CDCA, and deoxycholic acid (DCA) were increased in DHA/EPA-fed *db/db* mice, which was in accordance with the KEGG analysis of microbiome. Consistently, when we performed integrated pathway analysis by mapping all the differential annotated metabolites into biochemical pathways, multiple pathways were perturbed including ‘‘D-glutamine and D-glutamate metabolism’’ and ‘‘primary/secondary bile acid biosynthesis’’ (Fig. 3d, f, h, j). Intriguingly, glutamate and bile acid metabolism have been implicated in the pathology of metabolic syndrome.

### DHA and EPA rescue glutamate-induced β cell apoptosis

**Fig. 4:**
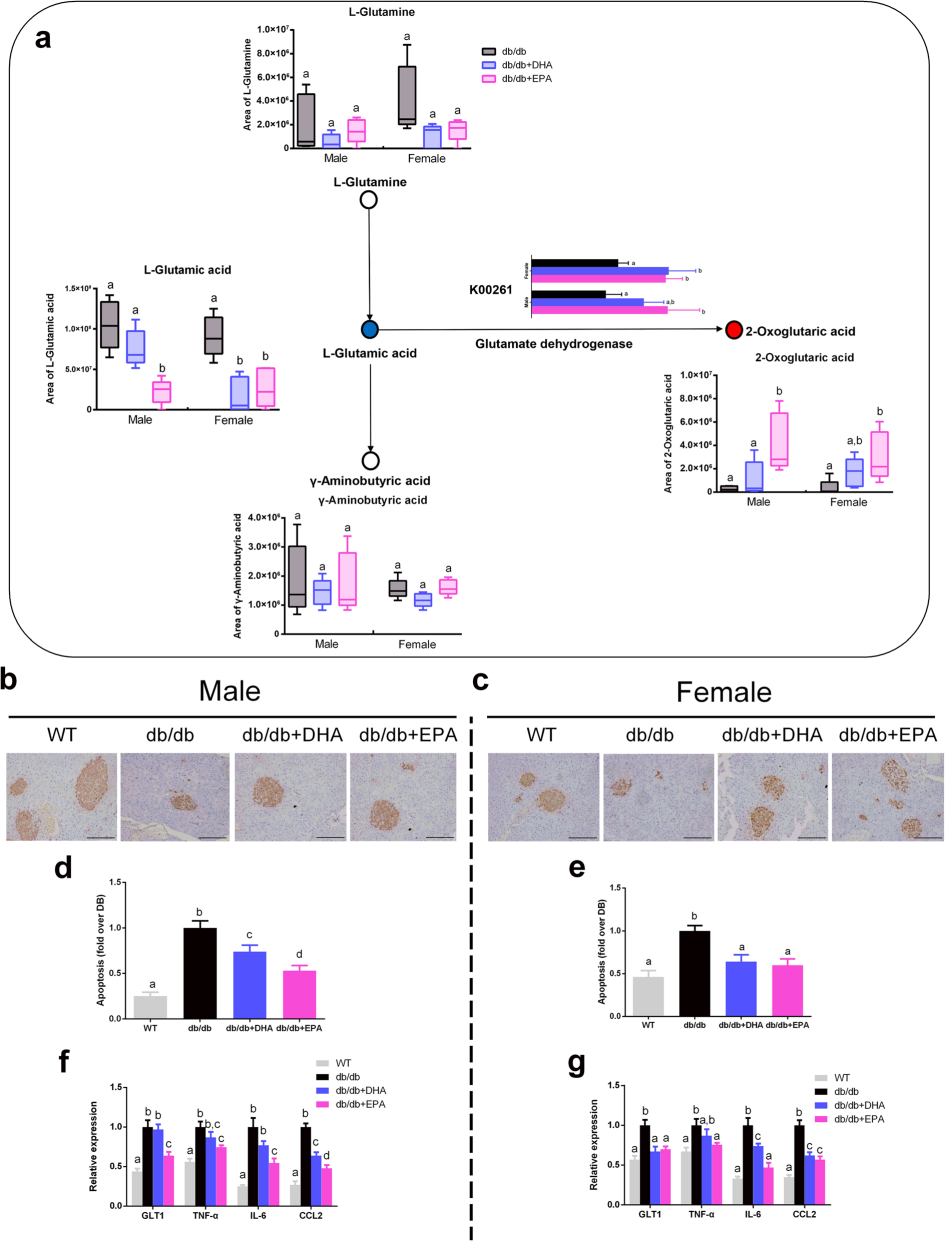
DHA/EPA accelerates glutamate degradation and rescues glutamate-induced β cell apoptosis. **a** Schematic overview of the differential metabolites involved in glutamate metabolism (*n* = 5 male and *n* = 5 female per group). Red circle represents up-regulated metabolites, blue circle represents downregulated metabolites, and open circle represents no change. Microbial gene encoding glutamate dehydrogenase (K00261) was upregulated by DHA/EPA. **b, c** Representative images of insulin staining in pancreases. Scale bar, 100 μm. **d, e** Apoptosis of β cell was determined by ELISA. Data are normalized to the *db/db* group (*n* = 6 male and *n* = 6 female per group). **f, g** mRNA expression of GLT1 and inflammatory cytokines in the pancreas. Groups with different superscript letters are significantly different (*P* < 0.05)

Given that DHA/EPA impacted glutamate metabolism which has been linked with metabolic disorders [33, 35], we further specifically compared the fecal content of l-glutamine, l-glutamate, γ-aminobutyric acid (GABA), and 2-oxoglutaric acid involved in the ‘‘D-glutamine and D-glutamate metabolism’’ pathway between the treatment groups (Fig. 4a). Although fecal levels of GABA and l-glutamine were not affected by DHA/EPA, l-glutamate level in fecal was significantly reduced in DHA-fed female mice and EPA-fed male and female mice. Conversely, the fecal 2-oxoglutaric acid level was elevated in EPA-receiving mice, while a trend toward a higher level of 2-oxoglutaric acid was detected in DHA-fed mice. In addition, we also observed similar changes in serum levels of l-glutamate and 2-oxoglutaric acid (Additional file 1: Figure S5a, b). Notably, microbial genes encoding glutamate dehydrogenase (K00261) which converses glutamate to 2-oxoglutaric acid was upregulated by DHA/EPA (Fig. 4a).

Chronic exposure to glutamic acid has been evidenced to exert a cytotoxic effect on β cell via activation of the glutamate transporter 1 (GLT1) [36]. Therefore, we then analyzed β cell apoptosis in the pancreas. Insulin immunohistochemistry showed that DHA/EPA rescued the β-cell death to some extent (Fig. 4b, c), which was further confirmed by a quantitative apoptosis assay (Fig. 4d, e). Moreover, we observed suppressed expression of GLT1 in DHA/EPA-fed mice with decreased expressions of inflammatory TNF-α, IL6, and CCL2 (Fig. 4f, g). Overall, EPA supplementation showed more pronounced changes compared with DHA. These findings indicate DHA/EPA supplementation enriched intestinal microbial species that possessed glutamate dehydrogenase to facilitate the degradation of glutamate and thereby prevented β cell apoptosis in *db/db* mice.

### DHA and EPA modulate bile acid metabolism and suppress hepatic gluconeogenesis

**Fig. 5:**
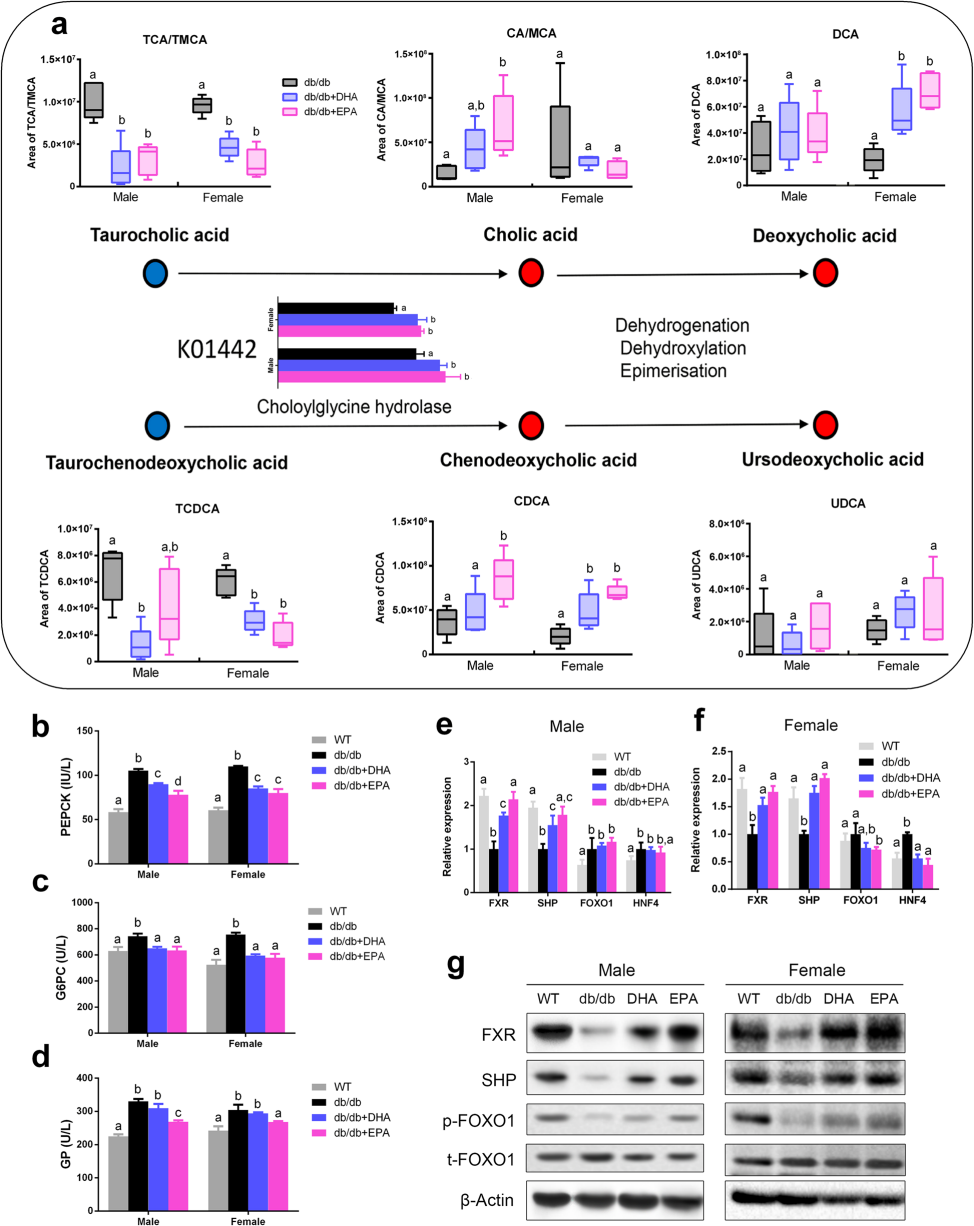
DHA/EPA influences bile acid metabolism and suppresses hepatic gluconeogenesis. **a** Schematic overview of the differential metabolites involved in bile acid metabolism (*n* = 5 male and *n* = 5 female per group). Red circle represents upregulated metabolites, blue circle represents downregulated metabolites, and open circle represents no change. Microbial gene encoding BSH (K01442) was upregulated by DHA/EPA. **b** Hepatic PEPCK concentrations. **c** Hepatic G6PC concentrations. **d** Hepatic GP concentrations. **e**, **f** Hepatic mRNA expression of FXR, SHP, FOXO1, and HNF4. **g** Representative hepatic immunoblots for FXR, SHP, p-FOXO1, and t-FOXO1. Groups with different superscript letters are significantly different (*P* < 0.05)

The gut microbiota-bile acid interaction plays a key role in regulating host lipid and glucose homeostasis [32]. We detected DHA/EPA enriched microbial genes encoding BSH (K01442) which deconjugated taurine-conjugated bile acids to form unconjugated bile acids (Fig. 5a). Consistently, we observed reduced fecal levels of TCA and TCDCA and increased levels of CA and CDCA in DHA/EPA-fed mice. Interestingly, DHA/EPA supplementation did not affect CA levels whereas substantially elevated DCA levels in female mice. Fecal UDCA level was not altered by DHA/EPA. Similar changes in these bile acids were also observed in serum (Additional file 1: Figure S5c–g).

Bile acids regulate hepatic glucose and lipid metabolism through modulating FXR [37]. We first observed that DHA/EPA administration significantly decreased the activity of hepatic PEPCK and G6PC involved in gluconeogenesis and EPA reduced GP involved in glycogenolysis (Fig. 5b–d). Next, we examined whether FXR-SHP-FOXO1 pathway was regulated. As anticipated, hepatic mRNA levels of FXR and SHP were promoted in DHA/EPA-fed mice (Fig. 5e, f). Western blot further verified that DHA/EPA supplementation increased protein expressions of FXR and SHP while induced the phosphorylation of FOXO1 (Fig. 5g). All these changes seemed stronger in EPA-fed mice compared with DHA-fed mice. Thus, these data suggest that DHA/EPA enriched BSH-positive gut microbiota and species that accelerated the production of unconjugated CA, CDCA, and secondary bile acid DCA, which activated FXR-SHP-FOXO1 pathway to inhibit hepatic gluconeogenesis.

FXR activation also regulates hepatic lipid homeostasis [32]. We also found decreased hepatic TG concentration despite unchanged TC, HDL-C, and LDL-C levels in DHA/EPA-fed mice (Additional file 1: Figure S6a–d). Liver sections of *db/db* mice showed DHA/EPA administration dramatically mitigated the extensive vacuolization, suggesting suppressed lipid accumulation (Additional file 1: Figure S6e, f). Subsequently, we observed mRNA expression of FASN associated with lipogenesis was consistently downregulated by DHA and EPA supplementation in male and female mice (Additional file 1: Figure S6g, h). Furthermore, DHA and EPA mitigated hepatic inflammation, which was indicated by immunofluorescence staining of F4/80 and decreased expressions of TLR4 and CCL2 (Additional file 1: Figure S6i–l).

### DHA and EPA promote SCFA production and beiging of WAT

**Fig. 6:**
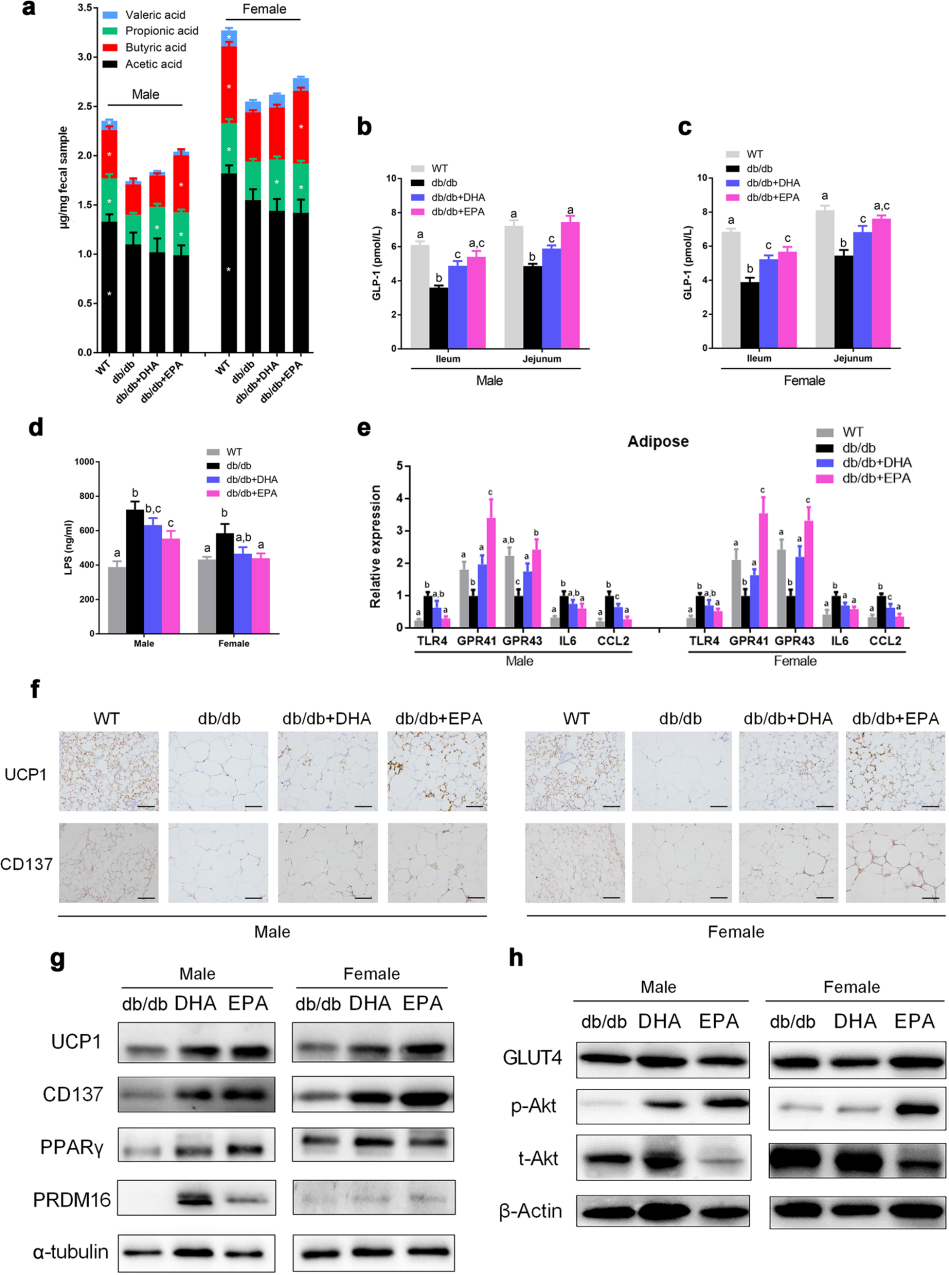
EPA and DHA promote SCFA production and beiging of WAT (*n* = 6 male and *n* = 6 female per group). **a** Individual SCFA levels in cecum content. **b**, **c** GLP-1 concentrations in small intestinal. **d** Serum LPS levels. **e** mRNA expression of TLR4, GPR41, GPR43, IL6, and CCL2 in WAT. **f** Representative images of UCP1 and CD137 staining in adipose tissues. Scale bar, 100 μm. **g** Representative adipose immunoblots for UCP1, CD137, PRDM16, and PPARγ. **h** Representative adipose immunoblots for GLUT4, p-AKT, and t-AKT. Graph bars with different superscript letters are significantly different (*P* < 0.05)

In light of significant changes in levels of propionic acid and butyric acid by DHA/EPA in UHPLC data of fecal content, we further assessed SCFA concentrations using GC. Results verified that propionate concentration was elevated by DHA and both propionate and butyrate were increased by EPA in male and female mice despite the unchanged levels of acetate and valerate (Fig. 6a). We also detected markedly increased concentration of GLP-1 by DHA and EPA administration in ileum and jejunum (Fig. 6b, c). SCFA production was proposed to stimulate the secretion of GLP-1 from L cells. Here, we verified that administration of propionate and butyrate could directly affect GLP-1 production in *db/db* mice (Additional file 1: Figure S7). Administration of butyrate significantly increased the production of GLP-1 in ileum and jejunum of *db/db* mice. Propionate supplementation showed a similar effect but was weaker than butyrate. EPA supplementation also significantly alleviated endotoxin in *db/db* mice (Fig. 6d). Accordingly, higher mRNA expressions of GPR41 and GPR43 and lower TLR4 expression was noted in the adipose tissue of DHA/EPA-treated mice (Fig. 6e). Moreover, mRNA expressions of proinflammatory IL6 and CCL2 in WAT were suppressed by DHA/EPA.

SCFA production was shown to attenuate inflammation and induce beiging of WAT [38, 39]. Subsequently, DHA and EPA supplementation markedly enhanced UCP1 and CD137 expressions as revealed by immunohistochemistry and western blot in inguinal WAT (Fig. 6f, g). Moreover, beiging program marker PRDM16 and PPARγ were also upregulated in DHA/EPA-fed mice (Fig. 6g). Finally, we observed evidently increased phosphorylation of Akt despite the unchanged expressions of GLUT4 in WAT, indicative of promoted GLUT4 translocation by DHA/EPA (Fig. 6h). Taken together, these findings suggest that DHA/EPA enhanced the generation of propionate and butyrate while alleviated endotoxin and inflammation and, subsequently, promoted WAT beiging and insulin signaling.

### DHA/EPA-altered gut microbes correlate with key metabolites

**Fig. 7:**
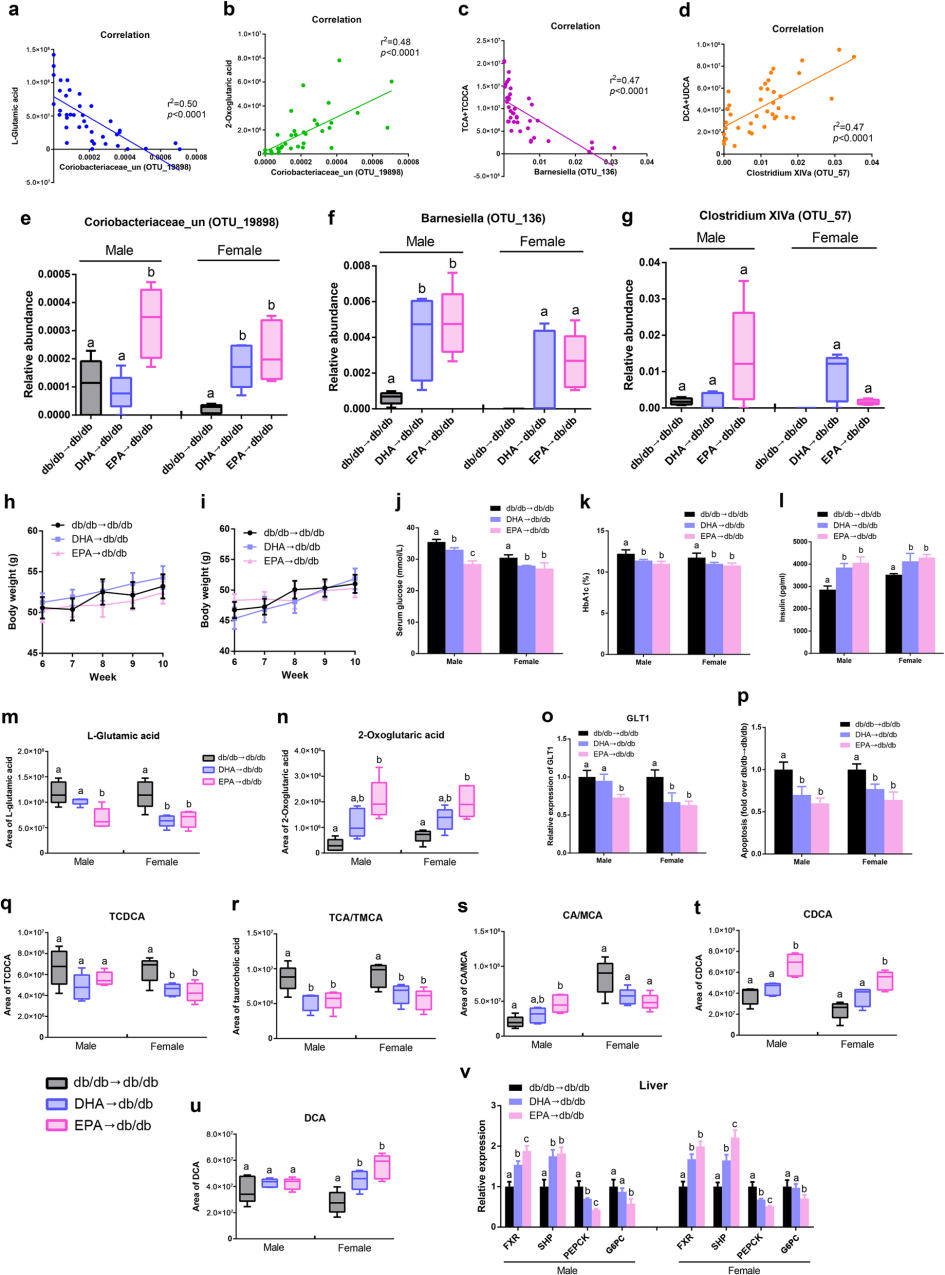
Transplantation of DHA/EPA-altered microbiota restores glucose homeostasis (*n* = 5 male and *n* = 5 female per group). **a–d** Linear regression analysis of fecal glutamate/bile acids with OTUs from genera identified in the microbe–metabolite network analysis. Fecal glutamate/bile acids show strong correlations with *Coriobacteriaceae* (OTU19898), *Barnesiella* (OTU136) and *Clostridium XlVa* (OTU57). **e–g** Relative abundance of *Coriobacteriaceae* (OTU19898), *Barnesiella* (OTU136), and *Clostridium XlVa* (OTU57) in *db/db* mice after fecal transplantation. Fecal transplantation from DHA/EPA-fed mice to *db/db* mice was performed as described in the “Methods” section. **h** Body weight curves for male mice during fecal transplantation. **i** Body weight curves for female mice during fecal transplantation. **j** Serum glucose levels after fecal transplantation. **k** Blood HbA1c levels. **l** Serum insulin levels. **m** Fecal l-glutamic acid levels. **n** Fecal 2-oxoglutaric acid levels. **o** mRNA expression of GLT1 in the pancreas. **p** Apoptosis of β cell normalized to the *db/db* group. **q** Fecal TCDCA levels. **r** Fecal TCA/TMCA levels. **s** Fecal CA/MCA levels. **t** Fecal CDCA levels. **u** Fecal DCA levels. **v** Hepatic mRNA expression of FXR, SHP, PEPCK, and G6PC. Groups with different superscript letters are significantly different (*P* < 0.05)

To determine the functional relationship between the DHA/EPA-altered gut microbes and metabolites, an inter-omic network was constructed using microbiome and metabolome and showed 250 strong correlations (|Spearman’s non-parametric rank correlation coefficient|> 0.6 and *p* < 0.05) between microbe (genera) and metabolite (Additional file 1: Figure S8). We identified genera *Coriobacteriaceae*, *Barnesiella*, and *Clostridium XlVa* showing strong correlations with fecal glutamate/bile acids from the network. Among these genera, the species *Coriobacteriaceae* (OTU19898) exhibited the highest negative correlation with glutamate acid (*r*^2^ = 0.50, *p* < 0.0001) and positive correlation with 2-oxoglutaric acid (*r*^2^ = 0.48, *p* < 0.0001) (Fig. 7a, b). In addition, the abundance of *Barnesiella* (OTU136) was inversely associated with taurine-conjugated bile acids (TCA + TCDCA) (*r*^2^ = 0.47, *p* < 0.0001) while *Clostridium XlVa* (OTU57) was positively related to fecal DCA level (*r*^2^ = 0.47, *p* < 0.0001) (Fig. 7c, d). These three key microbial species have been enriched in DHA/EPA-fed mice (Fig. 2c, d). Thus, these findings revealed the potential interactions between DHA/EPA-modulated microbial species and glutamate/bile acids.

### Gut microbiota orchestrates DHA/EPA-induced glucose homeostasis restoration

To investigate whether the DHA/EPA-induced intestinal microbial shift directly contributes to improved glucose metabolism, microbiota from DHA/EPA supplemented mice (referred as DHA/EPA-microbiota) were transplanted to microbiota-depleted *db/db* male and female mice. First, we validated the effect of antibiotic treatment on recipient *db/db* mice (Additional file 1: Figure S9). After transplantation with DHA/EPA-microbiota, recipient mice showed a similar pattern of gut microbial changes with DHA/EPA-fed mice (Additional file 1: Figure S10). Notably, the abundance of *Coriobacteriaceae* (OTU19898) and *Barnesiella* (OTU136) were remarkably elevated in mice transplanted with DHA/EPA-microbiota, and *Clostridium XlVa* (OTU57) was enriched in transplanted female mice (Fig. 7e–g), indicating the successful microbiota transplantation. Although the bodyweight gain was not affected, transplantation with DHA/EPA-microbiota significantly lowered the fasting glucose and Hb1Ac levels along with increased insulin level (Fig. 7h–l). In line with the enrichment of DHA/EPA-induced microbial species, we also detected decreased fecal levels of l-glutamic acid and increased 2-oxoglutaric acid. Accordingly, GLT1 expression was downregulated and β-cell death was mitigated in mice transplanted with DHA/EPA-microbiota (Fig. 7o, p). In addition, fecal taurine-conjugated bile acids were reduced while CA, CDCA, and DCA were increased in transplanted mice (Fig. 7q–u). Accordingly, hepatic mRNA expressions of FXR and SHP were promoted while PEPCK and G6PC expressions were inhibited by DHA/EPA-microbiota transplantation (Fig. 7v). Furthermore, transplanted mice also had higher fecal levels of propionate and butyrate, increased GLP-1 release in small intestinal and lower serum LPS concentration (Additional file 1: Figure S11a–c). As expected, we observed decreased TLR4 expression in WAT of EPA-microbiota transplanted mice and elevated expressions of GPR41 and GPR43 in DHA and EPA-microbiota transplanted mice (Additional file 1: Figure S11d). Moreover, adipose mRNA expressions of beiging markers including UCP1, CD137, PRDM16, and PPARγ were all significantly enhanced (Additional file 1: Figure S11e). All these alterations were more pronounced in EPA-microbiota-transplanted mice. Altogether, these results underscore the vital role of gut microbiota in the effects of DHA/EPA on preventing β cell apoptosis, suppressing hepatic gluconeogenesis, promoting GLP-1 secretion and inducing WAT beiging, which subsequently improved glucose homeostasis.

### DHA/EPA directly affect the growth of gut bacteria in vitro

**Fig. 8:**
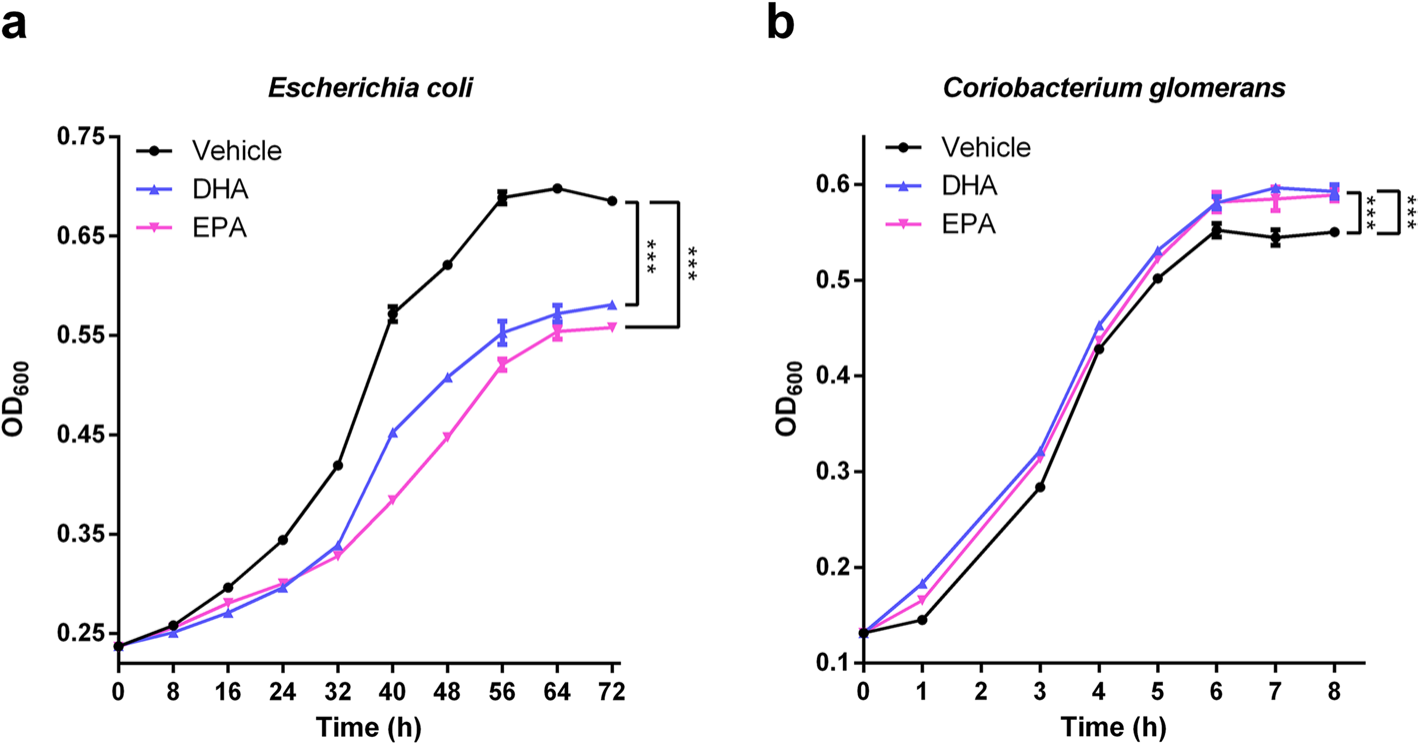
Effect of EPA and DHA on the growth of *Escherichia coli* and *Coriobacterium glomerans *in vitro*.* Strain *Escherichia coli* (**a**) was grown in LB medium and *Coriobacterium glomerans* (**b**) was grown in Gifu anaerobic medium. Media were added with DHA (200 µM), EPA (200 µM) or vehicle. Cell density of cultures was recorded at the indicated time points. Data are presented as the mean ± SEM (*n* = 6). *P* values were assessed by two-way ANOVA (*P < 0.05, **P < 0.01, ***P < 0.001)

To further evaluate the causal effect of DHA/EPA supplementation on the growth of specific gut microbiota, we selected pure cultures of *Escherichia coli* (Family Enterobacteriaceae) and *Coriobacterium glomerans* (Family Coriobacteriaceae) which were featured microbiological markers of *db/db* and *db/db* + DHA/EPA groups, respectively, and conducted in vitro intervention experiments. The bacterial growth media were added with DHA or EPA at 200 µM which was in a physiological range [40, 41]. As shown in Fig. 8a, supplementation of DHA and EPA significantly reduced the growth rates of *Escherichia coli* and the bacterial cell density for EPA group was lower than that of DHA group at the stationary growth phase, indicating a more potent antimicrobial activity of EPA. As regards to *Coriobacterium glomerans*, DHA and EPA similarly increased the bacterial cell density during the incubation period (Fig. 8b). These data demonstrated that DHA/EPA could directly inhibit the growth of *Escherichia coli* while promote *Coriobacterium glomerans*, which thereby drove the metabolic benefits.

## Discussion

In this study, DHA/EPA supplementation was found to dramatically attenuate hyperglycemia and IR in *db/db* mice. This amelioration was mediated by gut microbiome alterations and metabolites including glutamate, bile acids, and SCFAs, which led to relieved β cell apoptosis, suppressed hepatic gluconeogenesis, enhanced GLP-1 production, and WAT beiging. Overall, EPA displayed a more pronounced therapeutic effect through the interactions between gut and pancreas, liver, and adipose.

Although replacing dietary fat with n-3 PUFAs has been reported to effectively prevent the development of hyperglycemia in dietary obese rodents [42–47], few studies have investigated the possible therapeutic action of n-3 PUFAs on impaired glucose homeostasis. A previous study in rats found no protective effect of n-3 PUFAs supplementation on already established IR [48], whereas EPA administration was shown to minimize saturated fat induced IR and enhance fatty acid oxidation [16]. Here, we further demonstrated that DHA/EPA supplementation was able to attenuate hyperglycemia and restore impaired glucose homeostasis in *db/db* mice while the ameliorative effect was more potent for EPA. Similarly, the same low dose of EPA but not DHA was evidenced to protect against glucose intolerance in obese mice [14]. Together, these results suggest a promising role of EPA rather than DHA in diabetes treatment and management.

Previous studies have shown that fish oil containing EPA and DHA improved intestinal dysbacteriosis and influenced the gut microbiota content [10, 20, 21]. Compared with wild-type mice, *fat-1* transgenic mice which endogenously convert n-6 to n-3 PUFAs had less abundant *Ruminococcus* but more abundant *Akkermansia*, S24-7, and Tenericutes [49]. Here, we showed both DHA and EPA increased the diversity and modulated the composition of gut microbiota while the similarities and differences in their effects on gut microbiome were also documented. Notably, both DHA and EPA reduced the abundance of the LPS-containing Enterobacteriaceae [20] and some pathogenic bacteria, including *Staphylococcus*, *Streptococcus*, and *Klebsiella* [50–52]. It is believed that gram-negative Enterobacteriaceae can release LPS, resulting in systemic subclinical inflammation and thereby impairing insulin sensitivity [53]. *Streptococcus* infection could induce divergent phagocyte programmed cell death and cause robust inflammatory responses [52]. DHA and EPA elevated SCFA-producing *Prevotella* [54] and the family Coriobacteriaceae which was decreased in diabetic patients [55]. *Prevotella* is known to produce anti-inflammatory propionate, which could regulate the differentiation of anti-inflammatory Treg/Tr1 cells [54]. Additionally, DHA specifically enriched *Enterorhabdus* which was previously identified in non-diabetic lean animals [56], SCFA-producing *Alloprevotella* [57], and *Clostridium XlVa* which was depleted in inflammatory bowel diseases [58], while EPA specifically enriched anti-inflammatory *Barnesiella* [59] and *Mucispirillum* [60], *Clostridium lV* which has been negatively associated with obesity [61], *Allobaculum* negatively correlated with IR [62] and butyrate-producing *Eubacterium* [63] and *Intestinimonas* [64]. *Barnesiella* was correlated with several immunoregulatory cells and suggested to render the gut environment less prone to inflammation [59]. *Allobaculum* [65] and *Eubacterium* [66] induced by probiotics supplementation exerted beneficial physiological effects on obese animals, underscoring the probiotic potential of these species. Notably, beneficial bacteria *Bifidobacterium* and *Lactobacillus* were promoted by DHA and EPA respectively in female *db/db* mice. *Bifidobacterium* and *Lactobacillus* have been reported to improve gut integrity and counteract endotoxemia and inflammation [67–69]. These substantial shifts in gut community structure induced by DHA and EPA may maintain the intestinal health and impact microbial metabolites production.

KEGG analyses of gut microbiome and metabolome consistently demonstrated that glutamate metabolism and bile acid metabolism were the key metabolic pathways modified by DHA/EPA. Administration of glutamate has been shown to induce adiposity and impair glycemic control in rodents [70, 71]. Mechanically, the elevated glutamic acid level was found to induce pancreatic β cell apoptosis through GLT1 [36, 72]. Previous studies also revealed a link between gut microbiome and glutamate metabolism [73, 74]. A recent intervention study demonstrated that gavage with *B. thetaiotaomicron* which carried glutamate decarboxylase reduced plasma glutamate and ameliorated metabolic syndrome in diet-induced obese mice [33], indicating a promising role of targeting gut microbiota in obesity management via glutamate metabolism regulation. Here, we found l-glutamate level was substantially decreased and glutamate-induced β cell apoptosis was rescued especially by EPA. Correlation analysis further discovered the species *Coriobacteriaceae* (OTU19898) exhibited the highest negative correlation with l-glutamate acid and positive correlation with 2-oxoglutaric acid. The family Coriobacteriaceae has been suggested to play an important role in glucose metabolism regulation [75]. *Coriobacterium glomerans*, a species from Coriobacteriaceae, was found to possess glutamate dehydrogenase (K00261) which conversed glutamate to 2-oxoglutaric acid [76]. In vitro, we detected that DHA and EPA directly promoted the growth of *Coriobacterium glomerans.* Therefore, we speculate that the protective effect of DHA/EPA supplementation on β cell apoptosis was driven by *Coriobacteriaceae* (OTU19898) that possessed glutamate dehydrogenase to accelerate glutamate degradation.

The interaction between gut microbiota and bile acids plays a key role in regulating host metabolism [32]. Primary bile acids are initially synthesized in the liver, predominantly conjugated with taurine in mice, and subsequently deconjugated and metabolized by the intestinal microbiota into secondary bile acids, which affects FXR activation and signaling [32]. The primary bile acid CDCA is the strongest agonist for FXR followed by CA, the secondary bile acid DCA, and LCA [77], while the murine taurine-conjugated bile acids TMCA antagonize FXR [78]. Activation of FXR was shown to improve hyperlipidemia and hyperglycemia in *db/db* mice by repressing hepatic gluconeogenesis and increasing glycogen synthesis [79]. Furthermore, bile acids were evidenced to regulate hepatic gluconeogenic gene expression via the FXR-SHP-FOXO1 regulatory pathway [80]. In our study, we showed DHA/EPA supplementation elevated CDCA and CA whereas reduced TCA levels along with activated FXR-SHP-FOXO1 pathway and suppressed hepatic gluconeogenesis. Notably, the abundance of *Barnesiella* (OTU136) was inversely associated with taurine-conjugated bile acids, indicating *Barnesiella* (OTU136) produced BSH (K01442) activity which deconjugated taurine-conjugated bile acids to form unconjugated bile acids. Additionally, DCA level was positively related to *Clostridium XlVa* (OTU57) which could be capable of producing secondary bile acids via dehydrogenation, dehydroxylation, and isomerization processes [32]. Taken together, we reason that DHA/EPA enriched BSH-producing *Barnesiella* (OTU136) and *Clostridium XlVa* (OTU57) that promoted the production of unconjugated CA, CDCA, and secondary bile acid DCA, which inhibited hepatic gluconeogenesis via FXR-SHP-FOXO1 pathway.

The interplay between gut and adipose tissue [23, 24] affects white adipose inflammation which subsequently participates in the IR development [81]. We also noted DHA/EPA administration reduced endotoxemia and promoted SCFA production, mainly propionic and butyric acids, which could be attributed to reduced LPS-containing Enterobacteriaceae [20] and increased propionic/butyric acid-producing taxa *Prevotella* [54], *Alloprevotella* [57], *Clostridium XlVa* [58], *Eubacterium* [63], and *Intestinimonas* [64]*.* Propionic acid supplementation was reported to alleviate inflammation and improve glucose uptake in WAT from overweight adults [82]. Treatment of butyric acid also mitigated IR in mice [83] and attenuated inflammation through GPR41 and interaction with macrophages in 3T3-L1 adipocytes [84]. In contrast, increased production of acetic acid promoted metabolic syndrome in high fat diet (HFD)-fed mice via parasympathetic activation [85]. Accordingly, we detected increased expression of corresponding SCFA receptors (GPR41 and GPR43) in WAT and enhanced insulin signaling especially by EPA. Moreover, SCFAs supplementation was shown to promote beige adipogenesis, mitochondrial biogenesis, and FFA oxidation in HFD-induced obese mice by regulating GPRs [38]. Additionally, we showed that propionate and butyrate administration could directly increase GLP-1 production in *db/db* mice. Thus, the DHA/EPA-induced SCFAs production may contribute to the beneficial metabolic effect observed in our study.

To confirm the causal relationship between gut microbiota and the hypoglycemic effect of DHA/EPA, we transplanted microbiota from DHA/EPA-fed *db/db* mice into antibiotic-treated *db/db* mice fed with a normal diet for 4 weeks. Antibiotic-treated mice rather than germ-free mice were used in our study because germ-free mice have an underdeveloped immune system which may confound our analysis [86]. We showed that transplantation of DHA/EPA altered gut microbiota to recipient mice successfully counteracted hyperglycemia and enhanced insulin production, together with the enriched abundance of *Coriobacteriaceae* (OTU19898), *Barnesiella* (OTU136), and *Clostridium XlVa* (OTU57). Furthermore, we also observed similar changes in fecal metabolites including glutamate, bile acid and SCFAs, with corresponding changes in receptors expressions. Therefore, these data demonstrate DHA/EPA-altered intestinal flora mediate the protective effect for DHA/EPA and provide evidence for an important role of DHA/EPA-induced fecal metabolites in improving glucose homeostasis via crosstalk between gut and organs, including pancreas, liver, and adipose tissue.

Notably, EPA appeared to be more potent than DHA in ameliorating glucose metabolism in *db/db* mice. Possible explanations are as follows: we first observed that DHA and EPA differentially modulated antimicrobial peptide production in the gut which further led to the differential gut microbiome and metabolome changes. Notably, EPA had a more pronounced antimicrobial activity against LPS-containing *Escherichia coli* in vitro*.* Additionally, EPA was more capable of regulating bile acids, promoting propionic/butyric acid production and suppressing glutamate, especially in male mice, which led to more pronounced improvements in β cell apoptosis, hepatic gluconeogenesis, and WAT insulin signaling. We also noted sex differences in intestinal microbiome changes in response to DHA/EPA which could be attributed to sex hormones and sex differences in immune function [87]. In our study, we successfully revealed the metabolic pathway of EPA/DHA induced microbiota using 16S rDNA sequencing and PICRUSt and also provided direct causal evidence. Future researches using shotgun metagenome sequencing, the most solid approach to evaluate the functional profiles of microbial communities, may provide more biological insights.

Although no study has investigated the effect of DHA/EPA supplementation on gut microbiome in T2D patients, some case–control studies demonstrated that compared with non-diabetic controls, T2D patients had higher abundance of Enterobacteriaceae [53] but lower Coriobacteriaceae [75], which was also observed in *db/db* mice. Importantly, we showed that EPA/DHA could directly alter the growth of these microbiotas in vitro. Therefore, EPA/DHA supplementation is likely to impact the human gut microbiota in a similar way and future clinical studies are warranted to confirm this inference.

## Conclusion

In summary, integrated multi-omic analyses uncovered that DHA/EPA supplementation ameliorated glucose homeostasis in the context of diabetes by modulating gut microbiome and metabolome, which further prevented β cell apoptosis, suppressed hepatic gluconeogenesis and induced WAT beiging (Additional file 1: Figure S12). The study offers new insights into the gut microbiota-organs axis as a suitable target for treating metabolic perturbations and also suggests EPA may be a more efficient therapeutic dietary supplement than DHA for diabetic patients.

## Methods

Detailed methodologies are available in Additional file 2: Supplementary methods.

### Animals and diets

Four-week-old C57BL/KsJ-*db/db* mice and C57BL/6 mice were purchased from the Model Animal Research Center of Nanjing University (Nanjing, China) and were individually housed in ventilated cages in the animal facility of Zhejiang Chinese Medical University (Hangzhou, China) with a 12-h light/dark cycle, free access to food and water and standard conditions. The *db/db* mice were randomly assigned to one of three diet groups (12 mice for each group): the normal control diet (AIN93G; Research Diets, Inc., USA) (*db/db*); control diet supplemented with 1% (w/w) of DHA (purity > 99%; Larodan Fine Chemicals, Malmo, Sweden) (*db/db* + DHA); and control diet supplemented with 1% (w/w) of EPA (purity > 99%; Larodan Fine Chemicals) (*db/db* + EPA) for 10 weeks. A group of C57BL/6 J mice (WT) was also fed a control diet for 10 weeks as the positive control. The compositions of diets are shown in Additional file 1: Table S1. After the treatment, mice were euthanized and blood samples were collected. Tissues were carefully dissected and flash-frozen in liquid nitrogen.

### Indirect calorimetry

At the 9th week of treatment, metabolic parameters including oxygen consumption and respiratory exchange ratio (RER) were measured by an 8-cage animal monitoring system (PhenoMaster/LabMaster, Bad Homburg, Germany) at room temperature under 12-h day/night cycles.

### Glucose, insulin, and pyruvate tolerance tests

OGTT, ITT, and pyruvate tolerance test (PTT) were performed during the last week of the intervention. After overnight fasting, mice were orally gavaged with 66% glucose solution (OGTT, 3 g/kg BW) or injected i.p. with sodium pyruvate (PTT, 2 g/kg BW). For ITT, mice were injected i.p. with insulin (0.75 U/kg BW) after fasting for 6 h. Blood glucose levels in the blood samples from tail veins were measured immediately before (*t* = 0 min) and at selected time points using an Accu-Check glucose meter (Roche).

### Biochemical analyses

The serum glucose, TG, TC, high-density lipoprotein cholesterol, and low-density lipoprotein cholesterol were measured using a 7020 automatic biochemistry analyzer (Hitachi Ltd., Tokyo, Japan). The serum insulin, LPS, TNF-α and IL6, intestine GLP1, and hepatic PEPCK, G6PC, and GP levels were detected using commercial enzyme-linked immunosorbent assay kits (Cusabio Biotech Co. Ltd., Wuhan, Hubei, China) according to manufacturer’s protocol.

### Gene expression

Total RNA was extracted from the small intestine, WAT, liver, and pancreas using TRIzol. Subsequently, equal amounts of total RNA were reverse-transcribed into cDNA with the RT reagent kit (Takara Bio Inc., Shiga, Japan). The mRNA expression was quantified by a two-step qRT-PCR method [88]. Sequences of the used primers are provided in Additional file 1: Table S2. Expression was normalized to the housekeeping gene glyceraldehyde-3-phosphate dehydrogenase.

### Western blot

Total cellular protein from liver and WAT was extracted by lysing frozen tissues in RIPA lysis buffer containing inhibitors of protease and phosphatase. Total proteins were denatured, resolved by SDS-PAGE and then transferred to PVDF membranes (Millipore). The membranes were blocked using Tris-buffered saline Tween containing 5% bovine serum albumin at room temperature for 1 h and subsequently incubated with the primary antibodies against FXR, FOXO1, p-FOXO1, SHP, UCP1, CD137, PRDM16, PPARγ, GLUT4, p-Akt^Ser473^, or Akt overnight at 4 °C. The loading control was β-Actin or α-tubulin.

### Fecal microbiota transplantation

Fecal transplantation was performed in the last 4 weeks of the dietary intervention based on an established protocol [61]. During the 4 weeks, stools from the donors in different groups (*db/db*, *db/db* + DHA, and *db/db* + EPA) were collected daily under a laminar flow hood in sterile conditions. Every 100 mg fresh stools were resuspended in 1 ml of sterile saline and intensively mixed by vortexing. Subsequently, the mixture was centrifugated at 800 g for 3 min and the supernatant was used as the transplant material which was prepared on the same day of transplantation within 10 min before gavage. Recipient mice were 4-week-old *db/db* mice fed with a control diet for 6 weeks synchronized with the *db/db* group. Before fecal transplantation, their endogenous microbiota were depleted by oral gavage with 200 μl mixed antibiotic solution (ampicillin, 1 g/l; metronidazole, 1 g/l; vancomycin, 0.5 g/l; neomycin, 0.5 g/l) daily for 3 days. The recipient mice were fed with a control diet and treated with 200 μl transplant material daily by oral gavage for 4 weeks.

### Gut microbiome analysis

Gut microbiome analysis was performed using high-throughput 16S rDNA sequencing as described previously [89]. Briefly, DNA was extracted from fecal samples using the QIAamp Fast DNA Stool Mini Kit. DNA concentration and integrity were determined and the V3–V4 region of the bacterial 16S rRNA was amplified by PCR to construct an amplicon sequencing library. The amplicon was standardized and purified before being paired-end sequenced by an Illumina MiSeq platform. Raw sequencing data were subjected to filtration using Trimmomatic, FLASH, and QIIME software. Sequences were assigned to OTUs at 97% similarity. Representative sequences were chosen for each OTU and taxonomic data were then assigned to each representative sequence using Ribosomal Database Project (RDP) Classifier v.2.2, trained on the Silva database version 123. The OTU table was rarified and Chao 1 metric was calculated by QIIME to estimate α-diversity. Subsequently, the microbial community clustering (β-diversity) was estimated by principal coordinate analysis (PCoA). The linear discriminant analysis (LDA) effect size (LEfSe) analysis was used to identify differential taxa of biological relevance between groups. Non-parametric analysis was performed to assess significant differences in specific taxa between groups using the Kruskall-Wallis test followed by Mann–Whitney test.

### Microbial functional profiles prediction

The microbial functional profiles of DHA/EPA-altered gut microbiota was predicted using PICRUSt software (http://picrust.github.io/picrust). This method predicts the gene family abundance from the phylogenetic information with an estimated accuracy of 0.8. Predicted gene class abundances were analyzed at KEGG Orthology group levels 3 [90]. Results from PICRUSt were further analyzed using Statistical Analysis of Metagenomic Profiles (STAMP).

### Metabolomic profiling analysis

The sample preparation methods were described previously [91]. UHPLC-Q-Orbitrap-HRMS analyses were performed using a quadrupole-orbitrap mass spectrometer (Q-Exactive, Thermo Fisher Scientific, Waltham, MA, USA) equipped with Dionex 3000 Ultimate UHPLC and autosampler (Thermo Fisher Scientific). Each injected solution was separated with the Acquity UPLC HSS T3 column. The UHPLC analysis followed a gradient elution program. High-resolution mass spectrometry analyses were performed with a heat electrospray ionization (HESI) source under both positive ion mode and negative ion mode. The acquisition mode of quadrupole-orbitrap analyses was set to be the full MS/dd-MS2 (TopN) mode, which is a combination of full MS mode and dd-MS2 mode. The dd-MS2 mode employed an orbitrap resolution of 17,500 with a maximum latency time of 50 ms, high energy collisional dissociation stepped normalized collision energy of 20, 40, and 60, and isolation window of 1.5 m/z. Data analyses were performed using the Xcalibur 4.1 and Compound Discoverer 2.1 software (Thermo Fisher Scientific). The quality control (QC) samples were prepared by pooling of each extracted fecal or serum samples. The Compound Discovery software was used to process the batch normalization automatically using the data from QC samples. Based on the data from mzCloud (https://www.mzcloud.org) and Human Metabolome Database (HMDB) (http://www.hmdb.ca), the structural formula of metabolites were identified according to the accurate mass of their MS and tandem MS spectra. Part of metabolites was verified by standards available in our lab.

After normalization (by weight of fecal or serum samples), all the extracted UHPLC-Q-Orbitrap-HRMS ions were imported into SIMCA software (version 14.1, Umetrics, Umea, Sweden) for statistical analysis. Principle component analysis (PCA) and OPLS-DA with unit variance (UV) scaling were carried out to discriminate DHA/EPA-treated mice from control mice. Fold changes were calculated as mass response ratio between two arbitrary groups (*db/db* + DHA vs. *db/db* or *db/db* + EPA vs. *db/db*). The variable contribution of the OPLS-DA model was ranked by the variable importance in the projection (VIP). Metabolites passing the threshold of VIP > 1 were considered as significantly different between the DHA/EPA-fed and control mice groups, which were validated at a univariate level with *P* < 0.05 and fold change ≥ 1.2 or ≤ 0.8. All the annotated differential metabolites were uploaded to MetaboAnalyst (https://www.metaboanalyst.ca/) to identify perturbed pathways according to the KEGG pathway database (https://www.genome.jp/kegg/).

### Bacterial culture in vitro

*Escherichia coli* MG1655 was grown aerobically in LB medium and *Coriobacterium glomerans* ATCC 49,209 was grown anaerobically in Gifu anaerobic medium (GAM) at 37 °C. At mid-exponential phase of bacterial growth, cultures were added with DHA (200 µM), EPA (200 µM) or vehicle and incubated at 37 °C. The cell density of the cultures was monitored by recording OD_600_ values of samples taken at various time points until the stationary growth phase.

### Statistical analysis

All data were presented as mean ± SEM. Differences between two groups were analyzed by two-tailed Student’s *t* test. Differences between three or more groups were evaluated through one-way analysis of variance (ANOVA) followed by Tukey’s multiple comparison post-test. Two-way ANOVA was used for data from indirect calorimetry experiments. Heat maps were generated using STAMP. The Spearman’s rho non-parametric correlations between specific taxa and fecal metabolites were calculated using the SAS statistical package (version 9.4, SAS Institute). Gephi Graph Visualization and Manipulation software version 0.9.2 was used to visualize the network. *P* < 0.05 was considered statistically significant.

## Supplementary Information

**Additional file 1: Supplementary Tables and Figures**

**Additional file 2: Supplementary methods**

**Additional file 3.** Microbiome OTU

**Additional file 4.** Fecal metabolome

## Data Availability

OTU tables of gut microbiome and UHPLC-Q-Orbitrap-HRMS metabolomics data are included in this published article (Additional file 3 and Additional file 4). All 16S rRNA gene sequencing reads data has been deposited to the National Center for Biotechnology Information’s Sequence Read Archive under accession number PRJNA609364.

## Abbreviations

CA: Cholic acid
CD: Control diet
CDCA: Chenodeoxycholic acid
DCA: Deoxycholic acid
DHA: Docosahexaenoic acid
EPA: Eicosapentaenoic acid
FMT: Fecal microbiota transplant
GLP-1: Glucagon-like peptide-1
GPR: G-protein-coupled receptors
HFD: High-fat diet
IR: Insulin resistance
LDA: Linear discriminant analysis
LEfSe: Linear discriminant analysis effect size
LPS: Lipopolysaccharide
OTU: Operational taxonomic unit
PCoA: Principal coordinate analysis
PICRUSt: Phylogenetic Investigation of Communities by Reconstruction of Unobserved States
PUFA: Polyunsaturated fatty acid
SCFA: Short-chain fatty acid
T2D: Type 2 diabetes
TLR4: Toll-like receptor 4
WAT: White adipose tissue

## References

[1] International Diabetes Federation (2019). IDF diabetes atlas.

[2] Finlay BB (2020). Are noncommunicable diseases communicable?. Science.

[3] Saad MJA, Santos A, Prada PO (2016). Linking gut microbiota and inflammation to obesity and insulin resistance. Physiology.

[4] Liang X, Bushman Frederic D, FitzGerald GA (2014). Time in motion: The molecular clock meets the microbiome. Cell.

[5] Scott KP, Gratz SW, Sheridan PO, Flint HJ, Duncan SH (2013). The influence of diet on the gut microbiota. Pharmacol Res.

[6] Kennedy A, Martinez K, Chuang C-C, LaPoint K, McIntosh M (2008). Saturated fatty acid-mediated inflammation and insulin resistance in adipose tissue: Mechanisms of action and implications. J Nutr.

[7] Cani PD, Bibiloni R, Knauf C, Waget A, Neyrinck AM, Delzenne NM (2008). Changes in gut microbiota control metabolic endotoxemia-induced inflammation in high-fat diet–induced obesity and diabetes in mice. Diabetes.

[8] Buckley JD, Howe PRC (2009). Anti-obesity effects of long-chain omega-3 polyunsaturated fatty acids. Obes Rev.

[9] Calder PC (2006). n−3 Polyunsaturated fatty acids, inflammation, and inflammatory diseases. Am J Clin Nutr.

[10] Caesar R, Tremaroli V, Kovatcheva-Datchary P, Cani Patrice D, Bäckhed F (2015). Crosstalk between gut microbiota and dietary lipids aggravates WAT inflammation through TLR signaling. Cell Metab.

[11] Zhuang P, Shou Q, Wang W, He L, Wang J, Chen J (2018). Essential fatty acids linoleic acid and α-linolenic acid sex-dependently regulate glucose homeostasis in obesity. Mol Nutr Food Res..

[12] Bhaswant M, Poudyal H, Brown L (2015). Mechanisms of enhanced insulin secretion and sensitivity with n-3 unsaturated fatty acids. J Nutr Biochem.

[13] Neschen S, Morino K, Rossbacher JC, Pongratz RL, Cline GW, Sono S (2006). Fish oil regulates adiponectin secretion by a peroxisome proliferator–activated receptor-γ–dependent mechanism in mice. Diabetes.

[14] Pinel A, Pitois E, Rigaudiere J-P, Jouve C, De Saint-Vincent S, Laillet B (2016). EPA prevents fat mass expansion and metabolic disturbances in mice fed with a Western diet. J Lipid Res.

[15] Huber J, Löffler M, Bilban M, Reimers M, Kadl A, Todoric J (2007). Prevention of high-fat diet-induced adipose tissue remodeling in obese diabetic mice by n-3 polyunsaturated fatty acids. Int J Obes.

[16] Kalupahana NS, Claycombe K, Newman SJ, Stewart T, Siriwardhana N, Matthan N (2010). Eicosapentaenoic acid prevents and reverses insulin resistance in high-fat diet-induced obese mice via modulation of adipose tissue inflammation. J Nutr.

[17] Oh DY, Talukdar S, Bae EJ, Imamura T, Morinaga H, Fan W (2010). GPR120 is an omega-3 fatty acid receptor mediating potent anti-inflammatory and insulin-sensitizing effects. Cell.

[18] White PJ, Arita M, Taguchi R, Kang JX, Marette A (2010). Transgenic restoration of long-chain n-3 fatty acids in insulin target tissues improves resolution capacity and alleviates obesity-linked inflammation and insulin resistance in high-fat-fed mice. Diabetes.

[19] Martins AR, Crisma AR, Masi LN, Amaral CL, Marzuca-Nassr GN, Bomfim LHM (2018). Attenuation of obesity and insulin resistance by fish oil supplementation is associated with improved skeletal muscle mitochondrial function in mice fed a high-fat diet. J Nutr Biochem.

[20] Ghosh S, DeCoffe D, Brown K, Rajendiran E, Estaki M, Dai C (2013). Fish oil attenuates omega-6 polyunsaturated fatty acid-induced dysbiosis and infectious colitis but impairs LPS dephosphorylation activity causing sepsis. PloS One..

[21] Yu H-N, Zhu J, Pan W-S, Shen S-R, Shan W-G, Das UN (2014). Effects of fish oil with a high content of n-3 polyunsaturated fatty acids on mouse gut microbiota. Arch Med Res..

[22] Mujico JR, Baccan GC, Gheorghe A, Díaz LE, Marcos A (2013). Changes in gut microbiota due to supplemented fatty acids in diet-induced obese mice. Br J Nutr.

[23] Caesar R, Reigstad CS, Bäckhed HK, Reinhardt C, Ketonen M, Östergren Lundén G (2012). Gut-derived lipopolysaccharide augments adipose macrophage accumulation but is not essential for impaired glucose or insulin tolerance in mice. Gut.

[24] Rabot S, Membrez M, Bruneau A, Gérard P, Harach T, Moser M (2010). Germ-free C57BL/6J mice are resistant to high-fat-diet-induced insulin resistance and have altered cholesterol metabolism. FASEB J.

[25] Thomas RM, Jobin C (2020). Microbiota in pancreatic health and disease: the next frontier in microbiome research. Nat Rev Gastroenterol Hepatol.

[26] Albillos A, de Gottardi A, Rescigno M (2020). The gut-liver axis in liver disease: Pathophysiological basis for therapy. J Hepatol.

[27] Tsukumo DML, Carvalho-Filho MA, Carvalheira JBC, Prada PO, Hirabara SM, Schenka AA (2007). Loss-of-function mutation in Toll-Like Receptor 4 prevents diet-induced obesity and insulin resistance. Diabetes.

[28] Pingitore A, Chambers ES, Hill T, Maldonado IR, Liu B, Bewick G (2017). The diet-derived short chain fatty acid propionate improves beta-cell function in humans and stimulates insulin secretion from human islets in vitro. Diabetes Obes Metab.

[29] Yadav H, Lee J-H, Lloyd J, Walter P, Rane SG (2013). Beneficial metabolic effects of a probiotic via butyrate-induced GLP-1 hormone secretion. J Biol Chem.

[30] Layden BT, Angueira AR, Brodsky M, Durai V, Lowe WL (2013). Short chain fatty acids and their receptors: new metabolic targets. Transl Res.

[31] Selwyn FP, Csanaky IL, Zhang Y, Klaassen CD (2015). Importance of large intestine in regulating bile acids and glucagon-like peptide-1 in germ-free mice. Drug Metab Dispos.

[32] Wahlström A, Sayin Sama I, Marschall H-U, Bäckhed F (2016). Intestinal crosstalk between bile acids and microbiota and its impact on host metabolism. Cell Metab.

[33] Liu R, Hong J, Xu X, Feng Q, Zhang D, Gu Y (2017). Gut microbiome and serum metabolome alterations in obesity and after weight-loss intervention. Nat Med.

[34] Canfora EE, Meex RCR, Venema K, Blaak EE (2019). Gut microbial metabolites in obesity, NAFLD and T2DM. Nat Rev Endocrinol.

[35] He K, Du S, Xun P, Sharma S, Wang H, Zhai F (2011). Consumption of monosodium glutamate in relation to incidence of overweight in Chinese adults: China Health and Nutrition Survey (CHNS). Am J Clin Nutr.

[36] Di Cairano ES, Davalli AM, Perego L, Sala S, Sacchi VF, La Rosa S (2011). The glial glutamate transporter 1 (GLT1) is expressed by pancreatic beta-cells and prevents glutamate-induced beta-cell death. J Biol Chem.

[37] Shapiro H, Kolodziejczyk AA, Halstuch D, Elinav E (2018). Bile acids in glucose metabolism in health and disease. J Exp Med.

[38] Lu Y, Fan C, Li P, Lu Y, Chang X, Qi K (2016). Short chain fatty acids prevent high-fat-diet-induced obesity in mice by regulating G protein-coupled receptors and gut microbiota. Sci Rep.

[39] Jeremic N, Chaturvedi P, Tyagi SC (2017). Browning of white fat: Novel insight into factors, mechanisms, and therapeutics. J Cell Physiol.

[40] Zhuang P, Lu Y, Shou Q, Mao L, He L, Wang J (2019). Differential anti-adipogenic effects of eicosapentaenoic and docosahexaenoic acids in obesity. Mol Nutr Food Res..

[41] Kopf T, Schmitz G (2013). Analysis of non-esterified fatty acids in human samples by solid-phase-extraction and gas chromatography/mass spectrometry. J Chromatogr B Analyt Technol Biomed Life Sci.

[42] Flachs P, Rossmeisl M, Kopecky J (2014). The effect of n-3 fatty acids on glucose homeostasis and insulin sensitivity. Physiol Res.

[43] Flachs P, Mohamed-Ali V, Horakova O, Rossmeisl M, Hosseinzadeh-Attar MJ, Hensler M (2006). Polyunsaturated fatty acids of marine origin induce adiponectin in mice fed a high-fat diet. Diabetologia.

[44] Lanza IR, Blachnio-Zabielska A, Johnson ML, Schimke JM, Jakaitis DR, Lebrasseur NK (2013). Influence of fish oil on skeletal muscle mitochondrial energetics and lipid metabolites during high-fat diet. Am J Physiol Endocrinol Metab.

[45] Rossmeisl M, Jilkova ZM, Kuda O, Jelenik T, Medrikova D, Stankova B (2012). Metabolic effects of n-3 PUFA as phospholipids are superior to triglycerides in mice fed a high-fat diet: possible role of endocannabinoids. PloS One..

[46] Ruzickova J, Rossmeisl M, Prazak T, Flachs P, Sponarova J, Vecka M (2004). Omega-3 PUFA of marine origin limit diet-induced obesity in mice by reducing cellularity of adipose tissue. Lipids.

[47] Storlien LH, Jenkins AB, Chisholm DJ, Pascoe WS, Khouri S, Kraegen EW (1991). Influence of dietary fat composition on development of insulin resistance in rats: Relationship to muscle triglyceride and ω-3 fatty acids in muscle phospholipid. Diabetes.

[48] Podolin DA, Gayles EC, Wei Y, Thresher JS, Pagliassotti MJ (1998). Menhaden oil prevents but does not reverse sucrose-induced insulin resistance in rats. Am J Physiol Regul Integr Comp Physiol.

[49] Bidu C, Escoula Q, Bellenger S, Spor A, Galan M, Geissler A (2018). The transplantation of ω3 PUFA–altered gut microbiota of fat-1 mice to wild-type littermates prevents obesity and associated metabolic disorders. Diabetes.

[50] Younge N, Yang Q, Seed PC (2017). Enteral high fat-polyunsaturated fatty acid blend alters the pathogen composition of the intestinal microbiome in premature infants with an enterostomy. J Pediatr.

[51] Blin C, Passet V, Touchon M, Rocha EPC, Brisse S (2017). Metabolic diversity of the emerging pathogenic lineages of Klebsiella pneumoniae. Environ Microbiol.

[52] Tsatsaronis JA, Walker MJ, Sanderson-Smith ML (2014). Host responses to group a streptococcus: cell death and inflammation. PLoS Pathog..

[53] Chen Q, Ma X, Li C, Shen Y, Zhu W, Zhang Y (2021). Enteric phageome alterations in patients with type 2 diabetes. Front Cell Infect Microbiol..

[54] Li J, Sung CYJ, Lee N, Ni Y, Pihlajamäki J, Panagiotou G (2016). Probiotics modulated gut microbiota suppresses hepatocellular carcinoma growth in mice. Proc Natl Acad Sci U S A.

[55] Karlsson FH, Tremaroli V, Nookaew I, Bergström G, Behre CJ, Fagerberg B (2013). Gut metagenome in European women with normal, impaired and diabetic glucose control. Nature.

[56] Geurts L, Lazarevic V, Derrien M, Everard A, Van Roye M, Knauf C (2011). Altered gut microbiota and endocannabinoid system tone in obese and diabetic leptin-resistant mice: impact on apelin regulation in adipose tissue. Front Microbiol.

[57] Song JJ, Tian WJ, Kwok L-Y, Wang YL, Shang YN, Menghe B (2017). Effects of microencapsulated Lactobacillus plantarum LIP-1 on the gut microbiota of hyperlipidaemic rats. Br J Nutr.

[58] Berry D, Reinisch W (2013). Intestinal microbiota: a source of novel biomarkers in inflammatory bowel diseases?. Best Pract Res Clin Gastroenterol.

[59] Weiss GA, Chassard C, Hennet T (2014). Selective proliferation of intestinal Barnesiella under fucosyllactose supplementation in mice. Br J Nutr.

[60] Herp S, Brugiroux S, Garzetti D, Ring D, Jochum LM, Beutler M (2019). Mucispirillum schaedleri antagonizes Salmonella Virulence to protect mice against colitis. Cell Host Microbe.

[61] Chang C-J, Lin C-S, Lu C-C, Martel J, Ko Y-F, Ojcius DM (2015). Ganoderma lucidum reduces obesity in mice by modulating the composition of the gut microbiota. Nat Commun.

[62] Lam YY, Ha CWY, Hoffmann JMA, Oscarsson J, Dinudom A, Mather TJ (2015). Effects of dietary fat profile on gut permeability and microbiota and their relationships with metabolic changes in mice. Obesity.

[63] Louis P, Flint HJ (2017). Formation of propionate and butyrate by the human colonic microbiota. Environ Microbiol.

[64] Bui TPN, Shetty SA, Lagkouvardos I, Ritari J, Chamlagain B, Douillard FP (2016). Comparative genomics and physiology of the butyrate-producing bacterium Intestinimonas butyriciproducens. Environ Microbiol Rep.

[65] Kong C, Gao R, Yan X, Huang L, Qin H (2019). Probiotics improve gut microbiota dysbiosis in obese mice fed a high-fat or high-sucrose diet. Nutrition.

[66] Roberfroid M, Gibson GR, Hoyles L, McCartney AL, Rastall R, Rowland I (2010). Prebiotic effects: metabolic and health benefits. Br J Nutr.

[67] Guarner F, Perdigon G, Corthier G, Salminen S, Koletzko B, Morelli L (2007). Should yoghurt cultures be considered probiotic?. Br J Nutr.

[68] Bajaj JS, Heuman DM, Hylemon PB, Sanyal AJ, Puri P, Sterling RK (2014). Randomized clinical trial: lactobacillus gg modulates gut microbiome, metabolome and endotoxemia in patients with cirrhosis. Aliment Pharmacol Ther.

[69] Kaliannan K, Wang B, Li X-Y, Kim K-J, Kang JX (2015). A host-microbiome interaction mediates the opposing effects of omega-6 and omega-3 fatty acids on metabolic endotoxemia. Sci Rep.

[70] Hermanussen M, García AP, Sunder M, Voigt M, Salazar V, Tresguerres JAF (2006). Obesity, voracity, and short stature: the impact of glutamate on the regulation of appetite. Eur J Clin Nutr.

[71] Gralka E, Luchinat C, Tenori L, Ernst B, Thurnheer M, Schultes B (2015). Metabolomic fingerprint of severe obesity is dynamically affected by bariatric surgery in a procedure-dependent manner. Am J Clin Nutr.

[72] Banday VS, Lejon K (2017). Elevated systemic glutamic acid level in the non-obese diabetic mouse is Idd linked and induces beta cell apoptosis. Immunology.

[73] Ottosson F, Brunkwall L, Ericson U, Nilsson PM, Almgren P, Fernandez C (2018). Connection between BMI-related plasma metabolite profile and gut microbiota. J Clin Endocrinol Metab.

[74] Mar Rodríguez M, Pérez D, Javier Chaves F, Esteve E, Marin-Garcia P, Xifra G (2015). Obesity changes the human gut mycobiome. Sci Rep.

[75] Liu H, Zhang H, Wang X, Yu X, Hu C, Zhang X (2018). The family Coriobacteriaceae is a potential contributor to the beneficial effects of Roux-en-Y gastric bypass on type 2 diabetes. Surg Obes Relat Dis.

[76] Stackebrandt E, Zeytun A, Lapidus A, Nolan M, Lucas S, Hammon N (2013). Complete genome sequence of Coriobacterium glomerans type strain (PW2T) from the midgut of Pyrrhocoris apterus L. (red soldier bug). Stand Genomic Sci..

[77] Wahlström A, Kovatcheva-Datchary P, Ståhlman M, Bäckhed F, Marschall HU (2017). Crosstalk between bile acids and gut microbiota and its impact on farnesoid X receptor signalling. Digest Dis.

[78] Sayin Sama I, Wahlström A, Felin J, Jäntti S, Marschall H-U, Bamberg K (2013). Gut microbiota regulates bile acid metabolism by reducing the levels of tauro-beta-muricholic acid, a naturally occurring FXR antagonist. Cell Metab.

[79] Zhang Y, Lee FY, Barrera G, Lee H, Vales C, Gonzalez FJ (2006). Activation of the nuclear receptor FXR improves hyperglycemia and hyperlipidemia in diabetic mice. Proc Natl Acad Sci U S A.

[80] Yamagata K, Daitoku H, Shimamoto Y, Matsuzaki H, Hirota K, Ishida J (2004). Bile acids regulate gluconeogenic gene expression via small heterodimer partner-mediated repression of hepatocyte nuclear factor 4 and Foxo1. J Biol Chem.

[81] Caprio S, Perry R, Kursawe R (2017). Adolescent obesity and insulin resistance: Roles of ectopic fat accumulation and adipose inflammation. Gastroenterology.

[82] Al-Lahham SA, Roelofsen H, Rezaee F, Weening D, Hoek A, Vonk R (2012). Propionic acid affects immune status and metabolism in adipose tissue from overweight subjects. Eur J Clin Invest..

[83] Wang X, He G, Peng Y, Zhong W, Wang Y, Zhang B (2015). Sodium butyrate alleviates adipocyte inflammation by inhibiting NLRP3 pathway. Sci Rep.

[84] Ohira H, Fujioka Y, Katagiri C, Mamoto R, Aoyama-Ishikawa M, Amako K (2013). Butyrate attenuates inflammation and lipolysis generated by the interaction of adipocytes and macrophages. J Atheroscler Thromb.

[85] Perry RJ, Peng L, Barry NA, Cline GW, Zhang D, Cardone RL (2016). Acetate mediates a microbiome–brain–β-cell axis to promote metabolic syndrome. Nature.

[86] Hooper LV, Littman DR, Macpherson AJ (2012). Interactions between the microbiota and the immune system. Science.

[87] Bolnick DI, Snowberg LK, Hirsch PE, Lauber CL, Org E, Parks B (2014). Individual diet has sex-dependent effects on vertebrate gut microbiota. Nat Comm.

[88] Bustin SA, Benes V, Garson JA, Hellemans J, Huggett J, Kubista M (2009). The MIQE guidelines: minimum information for publication of quantitative real-time PCR experiments. Clin Chem.

[89] Ye J, Lv L, Wu W, Li Y, Shi D, Fang D (2018). Butyrate protects mice against methionine-choline-deficient diet-induced non-alcoholic steatohepatitis by improving gut barrier function, attenuating inflammation and reducing endotoxin levels. Front Microbiol.

[90] Langille MGI, Zaneveld J, Caporaso JG, McDonald D, Knights D, Reyes JA (2013). Predictive functional profiling of microbial communities using 16S rRNA marker gene sequences. Nat Biotechnol.

[91] Cesbron N, Royer AL, Guitton Y, Sydor A, Le Bizec B, Dervilly-Pinel G (2017). Optimization of fecal sample preparation for untargeted LC-HRMS based metabolomics. Metabolomics.

