# Supplementary material for: Eicosapentaenoic and docosahexaenoic acids attenuate hyperglycemia through the microbiome-gut-organs axis in db/db mice
Source: Microbiome. 2021 Sep 10;9:185. doi: 10.1186/s40168-021-01126-6 (PMC8434703; doi:10.1186/s40168-021-01126-6)
Supplement: Supplementary file 2 — Additional file 1: Supplementary Tables and Figures [file 40168_2021_1126_MOESM2_ESM.docx]

# Additional file 1

# Table S1. Diet composition of each animal group

| **Diet** | **Control diet** | |  | **DHA enriched** | | **EPA enriched** | |
| --- | --- | --- | --- | --- | --- | --- | --- |
| **Group** | ***db/db* or WT** | |  | ***db/db*+DHA** | | ***db/db*+EPA** | |
|  | gm% | *kcal%* |  | gm% | *kcal%* | gm% | *kcal%* |
| Protein | 20 | *20* |  | 20 | *20* | 20 | *20* |
| Carbohydrate | 64 | *64* |  | 64 | *64* | 64 | *64* |
| Fat | 7 | 16 |  | 7 | 16 | 7 | 16 |
| Total |  | *100* |  |  | *100* |  | *100* |
| kcal/gm | 4.00 |  |  | 4.00 |  | 4.00 |  |
|  |  |  |  |  |  |  |  |
| **Ingredient** | **gm** | ***kcal*** |  | **gm** | ***kcal*** | **gm** | ***kcal*** |
| Casein | 200 | *800* |  | 200 | *800* | 200 | *800* |
| L-Cystine | 3 | *12* |  | 3 | *12* | 3 | *12* |
|  |  |  |  |  |  |  |  |
| Corn Starch | 397.486 | *1590* |  | 397.486 | *1590* | 397.486 | *1590* |
| Maltodextrin 10 | 132 | *528* |  | 132 | *528* | 132 | *528* |
| Sucrose | 100 | *400* |  | 100 | *400* | 100 | *400* |
| Cellulose, BW200 | 50 | *0* |  | 50 | *0* | 50 | *0* |
|  |  |  |  |  |  |  |  |
| Soybean Oil | 70 | *630* |  | 60 | *540* | 60 | *540* |
| EPA (unesterified) | 0 | *0* |  | 0 | *0* | 10 | *90* |
| DHA (unesterified) | 0 | *0* |  | 10 | *90* | 0 | *0* |
|  |  |  |  |  |  |  |  |
| t-Butylhydroquinone | 0.014 | *0* |  | 0.014 | *0* | 0.014 | *0* |
| Mineral Mix S10022G | 35 | *0* |  | 35 | *0* | 35 | *0* |
| Vitamin Mix V10037 | 10 | *40* |  | 10 | *40* | 10 | *40* |
| Choline Bitartrate | 2.5 | *0* |  | 2.5 | *0* | 2.5 | *0* |
| **Total** | **1000** | ***4000*** |  | **1000** | ***4000*** | **1000** | ***4000*** |

| **Fatty acid profile (% of total fatty acids)** | **Control diet** | | **DHA enriched** | **EPA enriched** |
| --- | --- | --- | --- | --- |
| C6:0 | 0.9 |  | 0.8 | 0.8 |
| C7:0 | 0.2 |  | 0.2 | 0.2 |
| C8:0 | 0.0 |  | 0.0 | 0.0 |
| C10:0 | 0.1 |  | 0.1 | 0.1 |
| C12:0 | 0.1 |  | 0.1 | 0.1 |
| C14:0 | 0.2 |  | 0.2 | 0.2 |
| C15:0 | 0.1 |  | 0.1 | 0.1 |
| C16:0 | 11.4 |  | 9.7 | 9.7 |
| C18:0 | 4.2 |  | 3.4 | 3.4 |
| C20:0 | 0.4 |  | 0.4 | 0.4 |
| C22:0 | 0.5 |  | 0.4 | 0.4 |
| C24:0 | 0.1 |  | 0.1 | 0.1 |
| **Total SFAs** | 18.3 |  | 15.5 | 15.5 |
| C16:1 | 0.1 |  | 0.1 | 0.1 |
| C18:1 | 20.6 |  | 17.4 | 17.5 |
| C19:1 | 0.3 |  | 0.3 | 0.3 |
| C20:1 | 0.2 |  | 0.1 | 0.1 |
| C22:1 | 0.1 |  | 0.1 | 0.1 |
| **Total MUFAs** | 21.3 |  | 18.0 | 18.0 |
| C18:2 n6 | 53.9 |  | 45.8 | 45.7 |
| C18:3 n6 | 0.1 |  | 0.1 | 0.1 |
| C20:2 n6 | 0.2 |  | 0.2 | 0.2 |
| **Total n-6 PUFAs** | 54.2 |  | 46.1 | 46.0 |
| C18:3 n3 | 6.2 |  | 5.3 | 5.3 |
| C20:5 n3 | 0.0 |  | 15.2 | 0.0 |
| C22:6 n3 | 0.0 |  | 0.0 | 15.2 |
| **Total n-3 PUFAs** | 6.2 |  | 20.5 | 20.5 |
| **n6/n3** | 8.7 |  | 2.2 | 2.2 |

SFA, Saturated fatty acid; MUFA, Monounsaturated fatty acid; PUFA, Polyunsaturated fatty acid

# Table S2. Primers used for quantitative RT-PCR

| Gene | Sequence (5’ to 3’) | |
| --- | --- | --- |
| *GAPDH* | Forward | AGGTCGGTGTGAACGGATTTG |
|  | Reverse | TGTAGACCATGTAGTTGAGGTCA |
| *UCP1* | Forward | AGTACCCAAGCGTACCAAGC |
|  | Reverse | GACCCGAGTCGCAGAAAAGA |
| *CD137* | Forward | CTCCAGCATAGGTGGACAGC |
|  | Reverse | CGTTGTGGGTAGAGGAGCAA |
| *PRDM16* | Forward | ATCGCCAACAGCGAGATGAA |
|  | Reverse | GATCTCAGGCCGTTTGTCCA |
| *PPARγ* | Forward | CCACCAACTTCGGAATCAGCT |
|  | Reverse | TTTGTGGATCCGGCAGTTAAGA |
| *SREBP1C* | Forward | CCGAGATGTGCGAACTGGA |
|  | Reverse | ATAGGGGGCGTCAAACAGG |
| *FASN* | Forward | TTGACGGCTCACACACCTAC |
|  | Reverse | ACAGCCTGGGGTCATCTTTG |
| *ACC* | Forward | GTTCAGAGAGTTCACCCAGCA |
|  | Reverse | AACTAGGAACGTAAGTCGCCG |
| *CPT1a* | Forward | CTCCGCTCGCTCATTCCG |
|  | Reverse | TGTGAACTGGAAGGCCACAG |
| *Fabp1* | Forward | TGCGAACTGGAGACCATGAC |
|  | Reverse | TCACCTTCCAGCTTGACGAC |
| *TLR-4* | Forward | TATCCAGGTGTGAAATTGAAACAATT |
|  | Reverse | GGGTTTCCTGTCAGTATCAAGTTTG |
| *TNF-α* | Forward | CCCAGACCCTCACACTCAGATC |
|  | Reverse | GCCACTCCAGCTGCTCCTC |
| *IL-6* | Forward | TGATGGATGCTACCAAACTGGA |
|  | Reverse | GGTACTCCAGAAGACCAGAGG |
| *CCL2* | Forward | AGGTGTCCCAAAGAAGCTGTAG |
|  | Reverse | TGTCTGGACCCATTCCTTCTTG |
| *GLT1* | Forward | ACAATATGCCCAAGCAGGTAGA |
|  | Reverse | CTTTGGCTCATCGGAGCTGA |
| *GPR41* | Forward | AGTGCCAGTTGTCCAATACTCT |
|  | Reverse | AAGAAGCTTGTCCCCATGGTC |
| *GPR43* | Forward | CAATCAGAAGACAGAAAAGGAGCTG |
|  | Reverse | GTCTGGGGTCATTCTCCTTGG |
| *Reg3g* | Forward | TGCTCCTTTCTCAGGTGCAA |
|  | Reverse | CAATAGGAGCCATAGGCACGG |
| *Pla2g2* | Forward | ACAGGATCGAGAATGGCACC |
|  | Reverse | TCACACTCGCATGTCTGCTT |
| *Defa* | Forward | TGTAGAAAAGGAGGCTGCAATAG |
|  | Reverse | AGAACAAAAGTCGTCCTGAGC |
| *PEPCK* | Forward | CGGATGGGCATATCTGTGCT |
|  | Reverse | AGGCCCAGTTGTTGACCAAA |
| *G6PC* | Forward | CCGGATCTACCTTGCTGCTC |
|  | Reverse | GCATTGTAGATGCCCCGGAT |
| *FXR* | Forward | TACCACTACAACGCGCTCAC |
|  | Reverse | ACACTTGTACACGGCGTTCT |
| *SHP* | Forward | GCACGATCCTCTTCAACCCA |
|  | Reverse | AGACTTCACACAGTGCCCAG |
| *FOXO1* | Forward | AGTGGATGGTGAAGAGCGTG |
|  | Reverse | GAAGGGACAGATTGTGGCGA |
| *HNF4* | Forward | TGCAAGGGGTTCTTCAGGAG |
|  | Reverse | TGTCTACCACACATTGTCGGC |

**

**

Figure S1. Effects of DHA and EPA on body fat and metabolic markers. The *db/db* mice were fed either a control diet (*db/db*), DHA enriched diet (*db/db*+DHA), or EPA enriched diet (*db/db*+DHA) for 10 weeks (*n* = 6 male and *n* = 6 female per group). (**a**) Food intake. (**b, c**) Mass of iWAT, gWAT and iBAT. (**d**) Serum total cholesterol levels. (**e**) Serum HDL-C levels. (**f**) Serum LDL-C levels. (**g**) Serum IL6 levels. (**h**) Serum TNF-α levels. Graphs show mean ± SEM. Data with different superscript letters are significantly different (*P*<0.05) using one-way ANOVA.


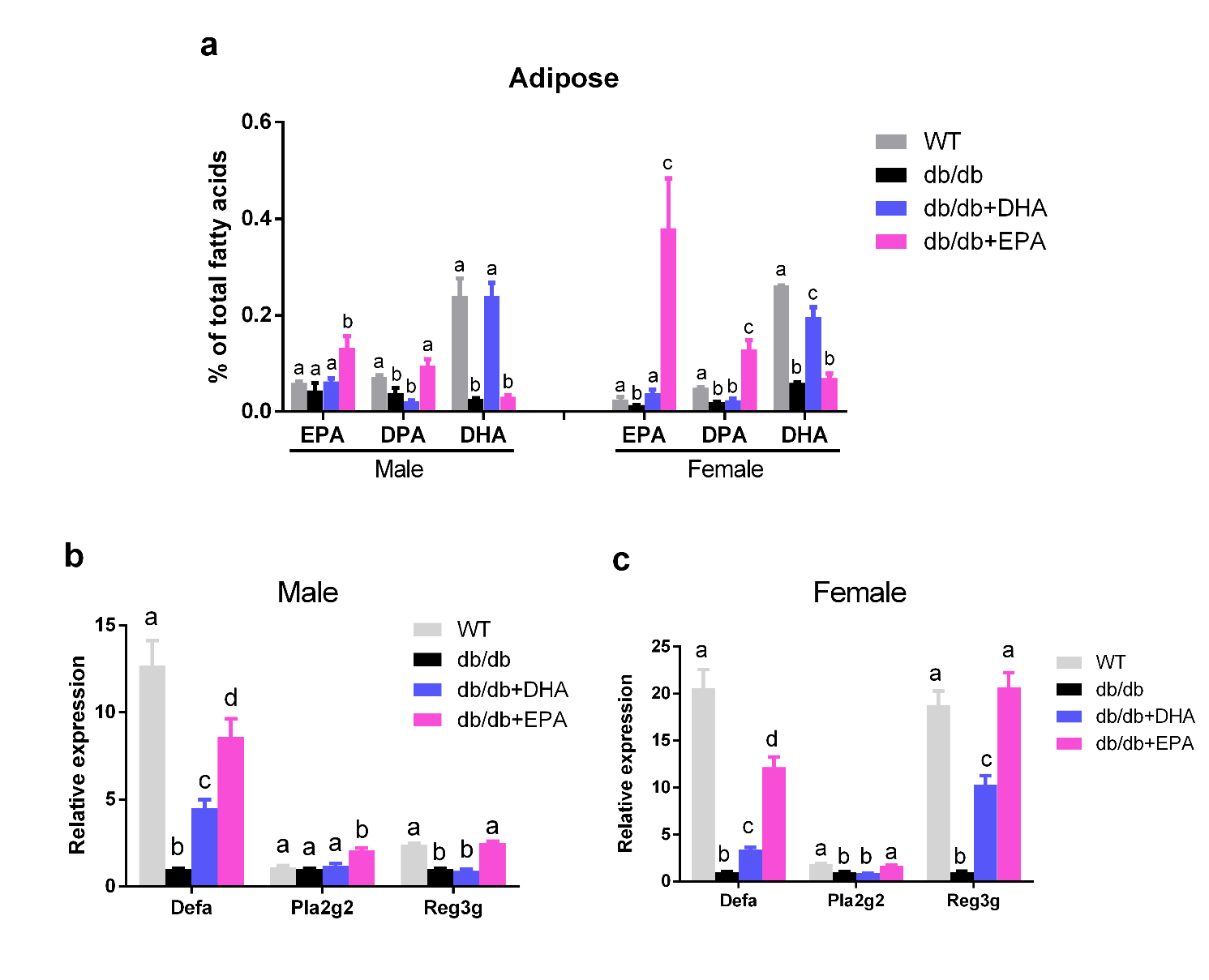


Figure S2. Effects of DHA/EPA supplementation on adipose marine n-3 fatty acids levels and intestinal antimicrobial peptide production in *db/db* mice. (a) Marine n-3 fatty acids levels in gonadal white adipose tissue. (b, c) mRNA expressions of α-defensin–Defa, Pla2g2a and Reg3g in the small intestine. Graphs show means ± SEM (*n*=6 male and *n*=6 female per group). Graph bars with different superscript letters are significantly different (*P*<0.05).


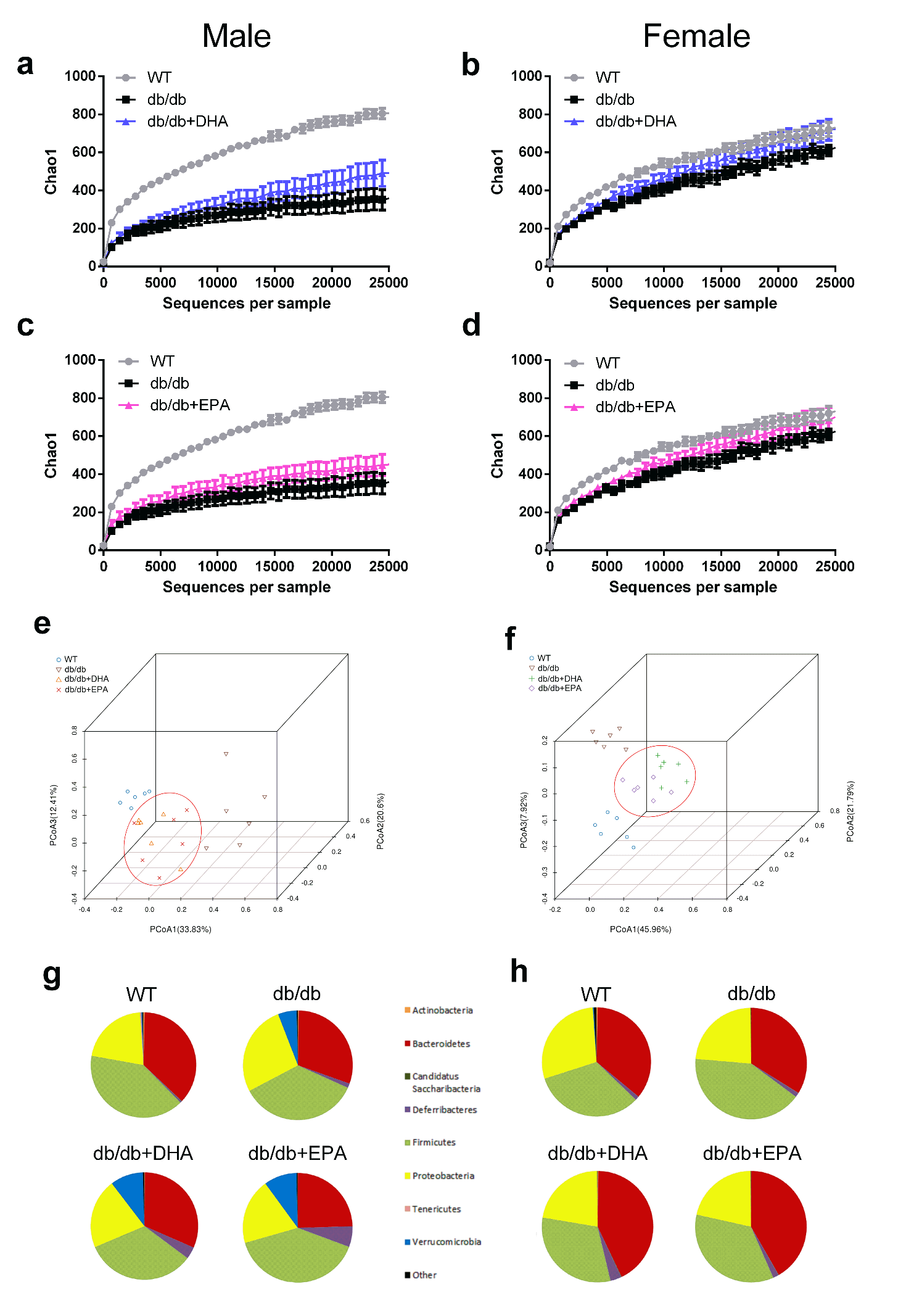


Figure S3. EPA and DHA differentially modulate the gut microbiome. Fecal microbiota composition in mice from different groups were analyzed using 16S rDNA sequencing. (**a-d**) Rarefaction curves for Chao1 (1-25000 sequences per sample). (**e**, **f**) PCoA based on unweighted UniFrac. (**g**, **h**) Pie charts of fecal microbiota composition at the phylum level.

**
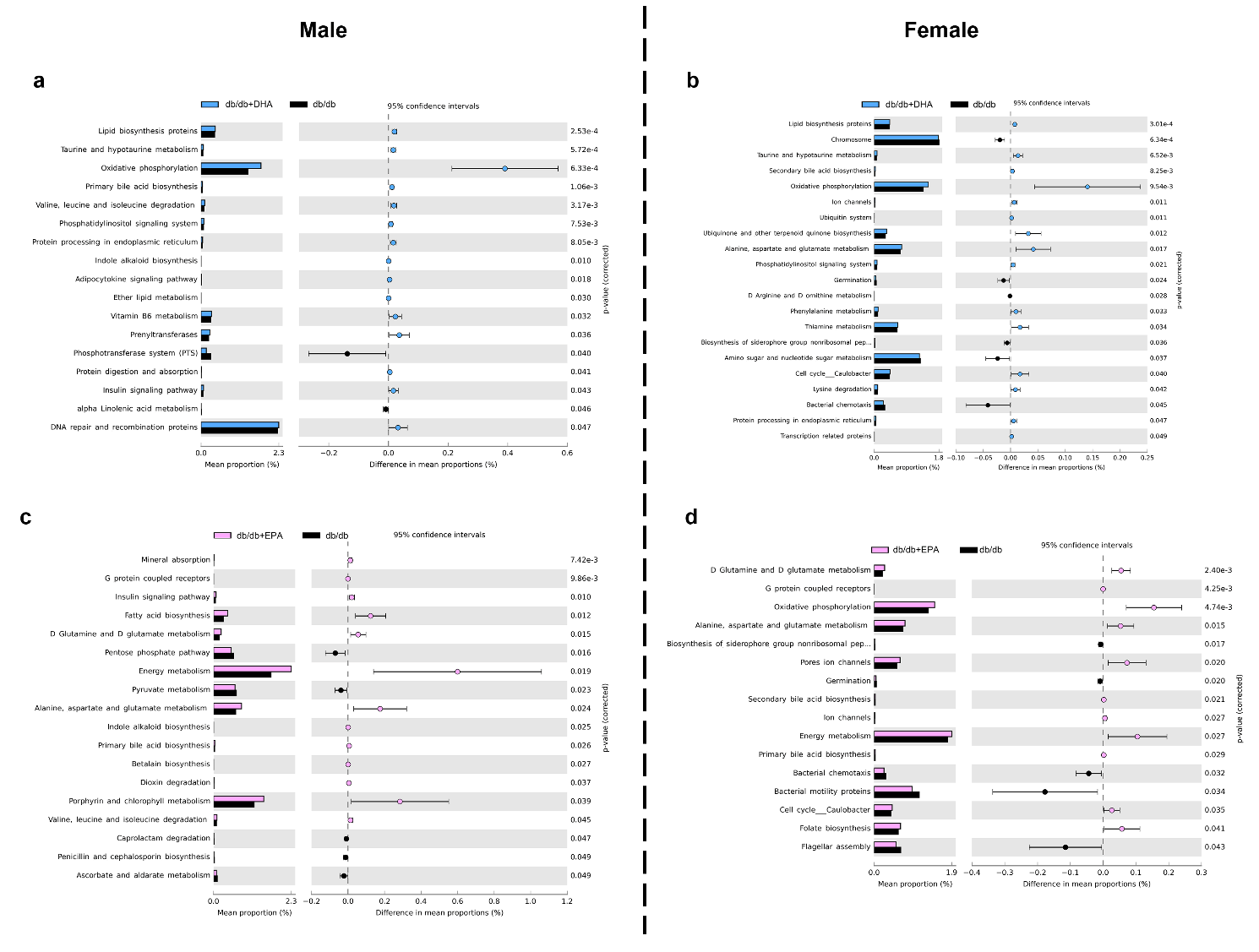
**

Figure S4. Metabolic functions within the microbiome altered by DHA/EPA. (a-d) Imputed metagenomic differences between DHA/EPA-fed and control mice groups in KEGG pathway maps. The relative abundance of metabolic pathways encoded in each imputed sample metagenome was analyzed using STAMP.


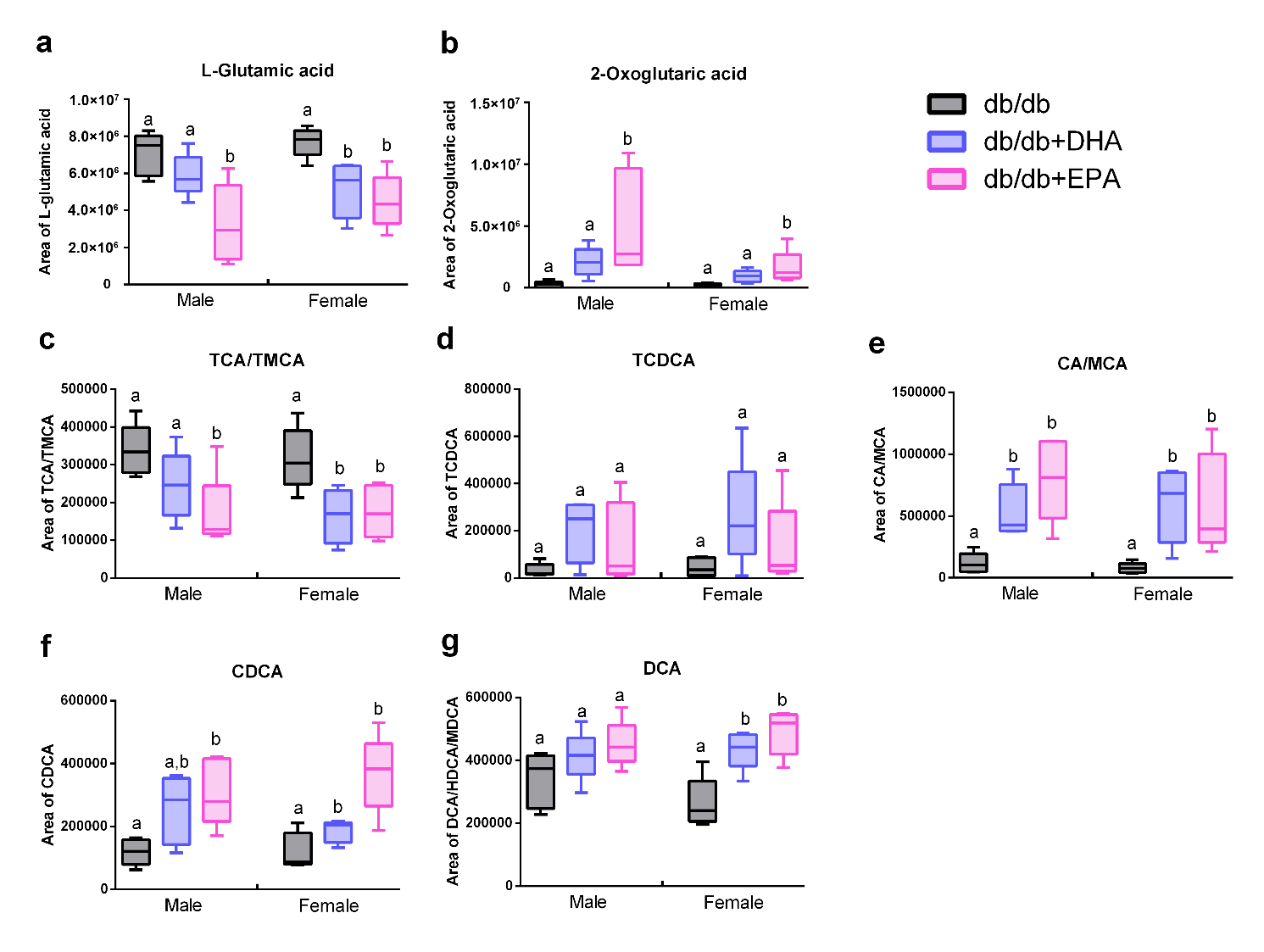


Figure S5. Changes in circulating glutamate and bile acids in DHA/EPA fed mice. Serum levels of L-glutamic acid (**a**), 2-Oxoglutaric acid (**b**), TCA/TMCA (**c**), TCDCA (**d**), CA/MCA (**e**), CDCA (**f**), and DCA (**g**) were detected using UHPLC-Q-Orbitrap-HRMS. *n* = 5 male and *n* = 5 female per group. Groups with different superscript letters are significantly different (*P*<0.05).


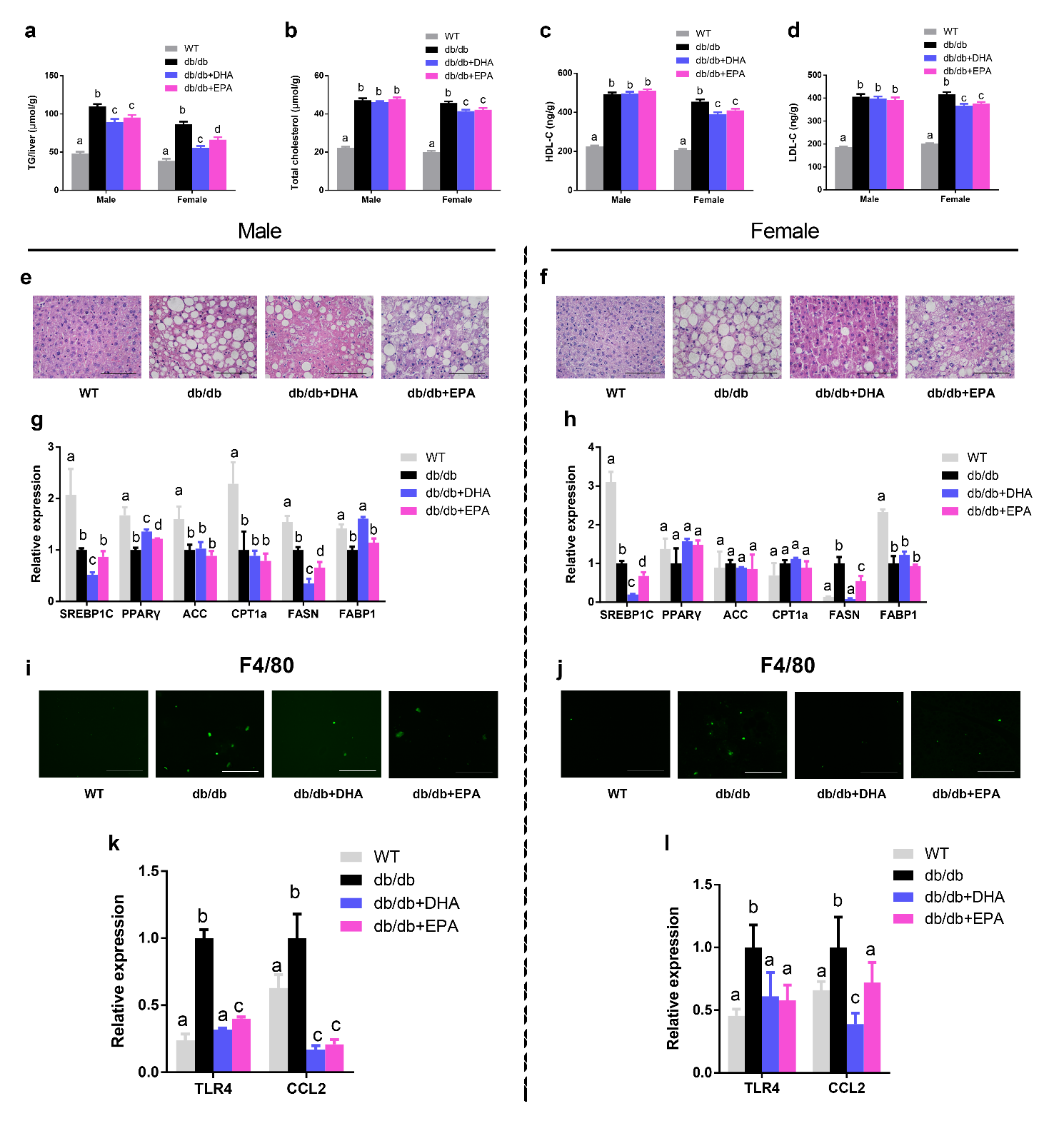


Figure S6. DHA and EPA supplementation improve hepatic lipid metabolism in *db/db* mice. (a-d) Hepatic levels of TG, TC, HDL-C, and LDL-C. (e, f) Representative photomicrographs of liver sections using H-E staining. Scale bar, 100 μm. (**g, h**) Hepatic mRNA expression of genes participating in hepatic TG metabolism. (**i, j**) F4/80 staining in livers. Scale bar, 100 μm. (**k, l**) Hepatic mRNA expression of TLR4 and CCL2. Graphs show means ± SEM (*n*=6 male and *n*=6 female per group). Graph bars with different superscript letters are significantly different (*P*<0.05).





Figure S7. Propionate and butyrate supplementation promoted the production of GLP-1 in *db/db* mice. Graphs show means ± SEM of GLP-1 concentrations in small intestinal (*n*=6 per group). Graph bars with different superscript letters are significantly different (*P*<0.05).


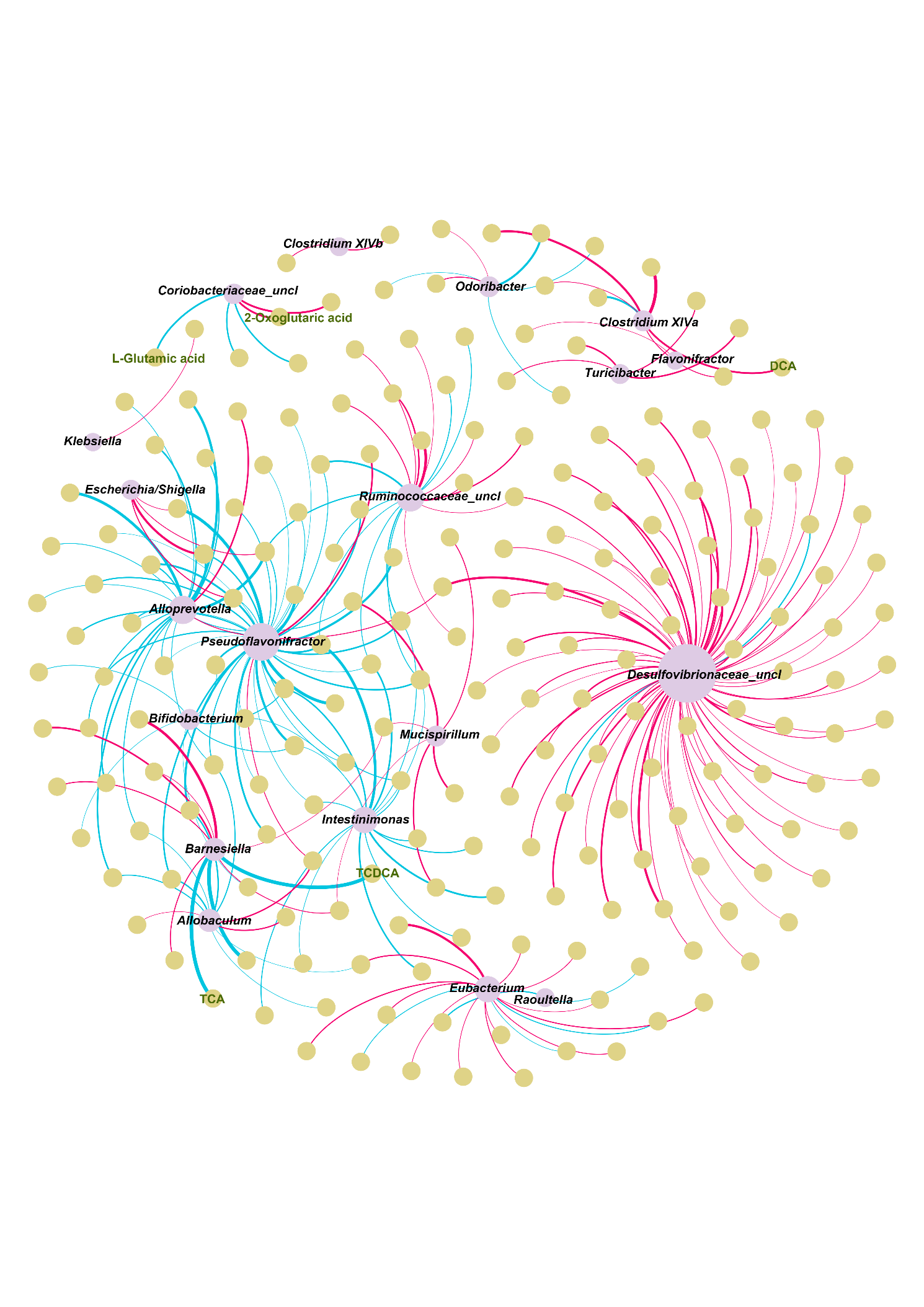


Figure S8. Metabolic network modeling and taxa-metabolite associations. An inter-omic network was constructed with 229 nodes (filled circles) representing microbes (pink) and fecal metabolites (brown). Correlations with |Spearman’s correlation coefficient| > 0.6 and *P* < 0.05 are shown. Edges represent 140 positive (red) and 110 negative (blue) correlations between microbe–metabolite pairs and edge width is proportional to the strength of association. Highlighted metabolites (green) are key glutamate/bile acids strongly associated with genera *Coriobacteriaceae*, *Barnesiella* or *Clostridium XlVa*.


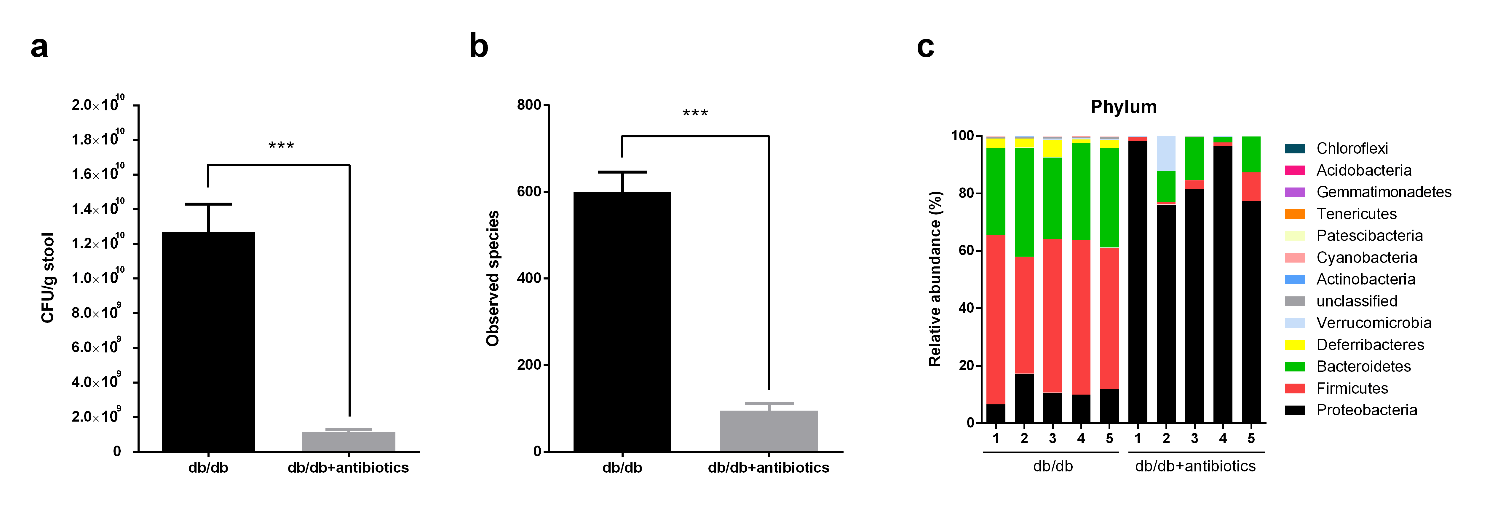


Figure S9. Analysis of gut microbiota in recipient *db/db* mice treated with mixed antibiotic solution. (**a**) Cecal content, CFU/g. (**b**) Observed species. (**c**) Bacterial taxonomic profiling in the phylum level of intestinal bacteria. The antibiotic treatment substantially reduced the global density of the gut microbiota by approximately 90%, dramatically decreased α-diversity (observed species), and also altered its relative composition. Data are presented as the mean ± SEM (n=5). *P* values were assessed by two-tailed Student’s t-test (**P*<0.05, ***P*<0.01, ****P*<0.001).


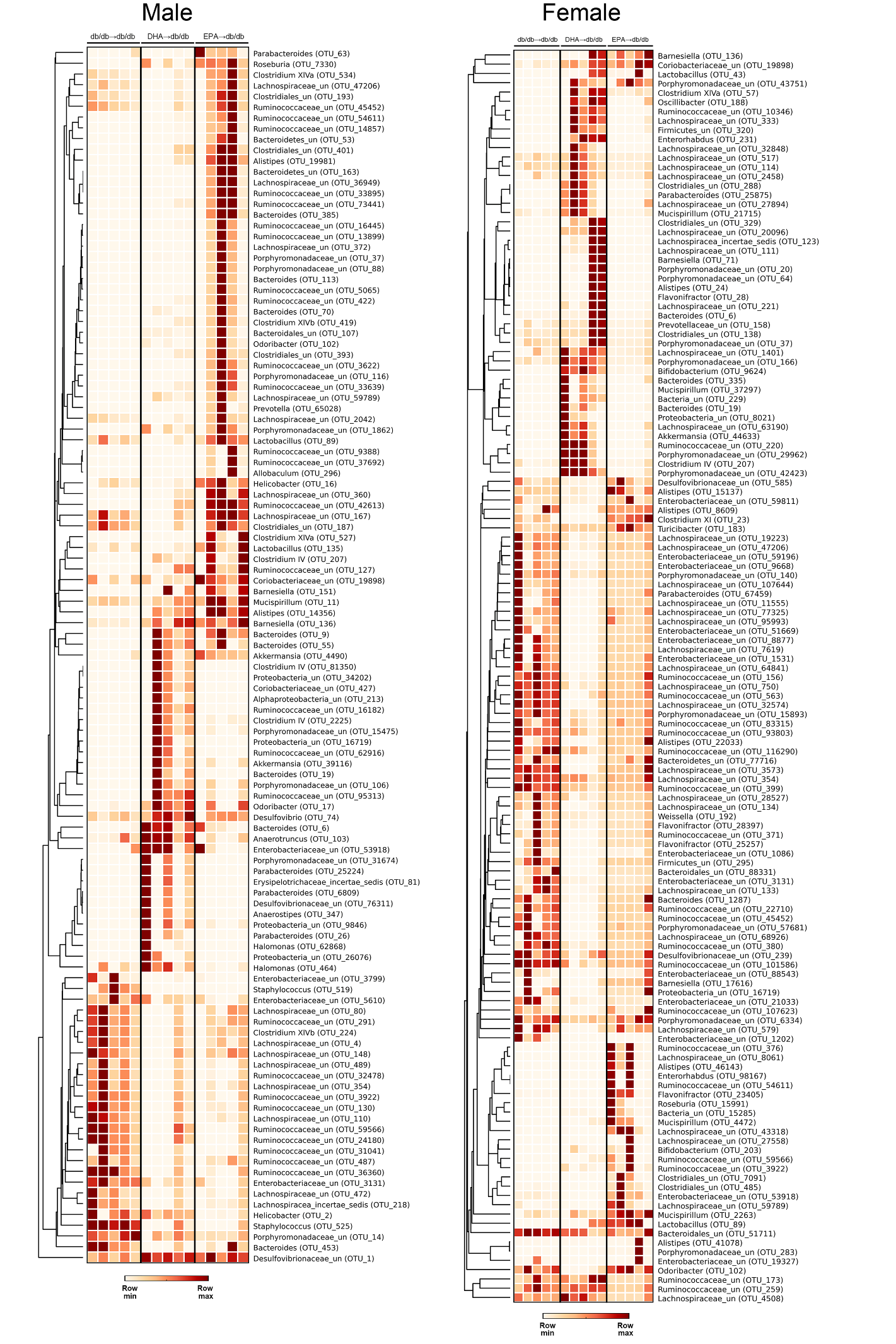


Figure S10. Analysis of gut microbiota following fecal transplantation. Fecal microbiota composition in transplanted mice were analyzed using 16S rDNA sequencing. Heatmaps showing OTUs with significantly different relevant abundances between three treatment groups (as determined by Kruskall-Wallis test followed by Mann-Whitney test). Each row represents an OTU labeled by the lowest taxonomic description and OTU ID, normalized to the row maximum.

**

**

Figure S11. Transplantation of DHA/EPA-altered microbiota promotes SCFA production and WAT beiging. Fecal transplantation from DHA/EPA-fed mice to *db/db* mice was performed as described in the Methods. (**a**) Individual SCFA levels in cecum content after transplantation. (**b**) GLP-1 concentrations in small intestinal. (**c**) Serum LPS levels. (**d, e**) mRNA expression of TLR4, GPR41, GPR43, UCP1, CD137, PRDM16 and PPARγ in WAT. Graph bars with different superscript letters are significantly different (P<0.05).

**
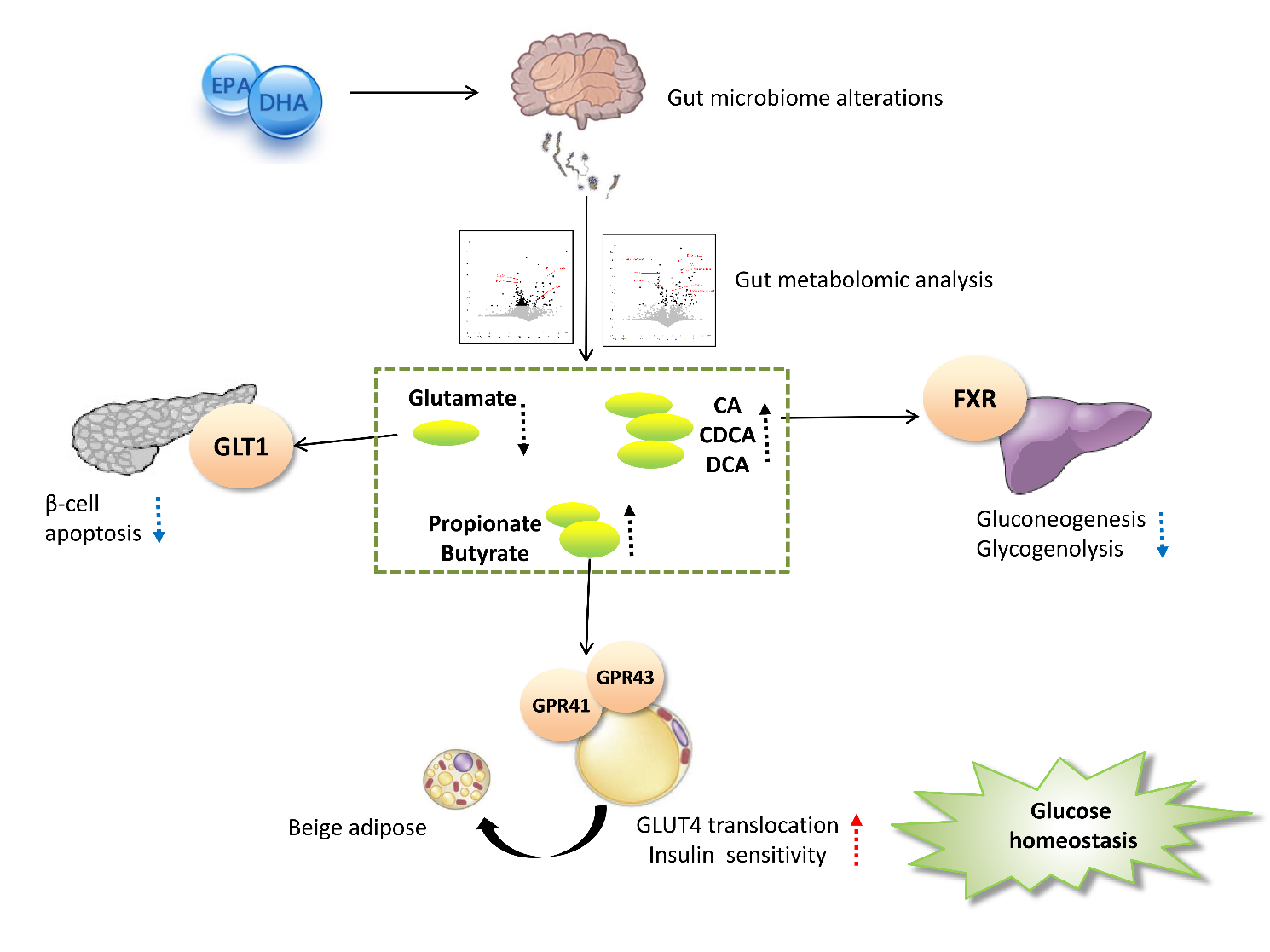
**

Figure S12. Summary of findings from the present study. DHA/EPA attenuates hyperglycemia and insulin resistance in *db/db* mice through alterations in gut microbiome and metabolites linking gut to adipose, liver and pancreas.

**­­
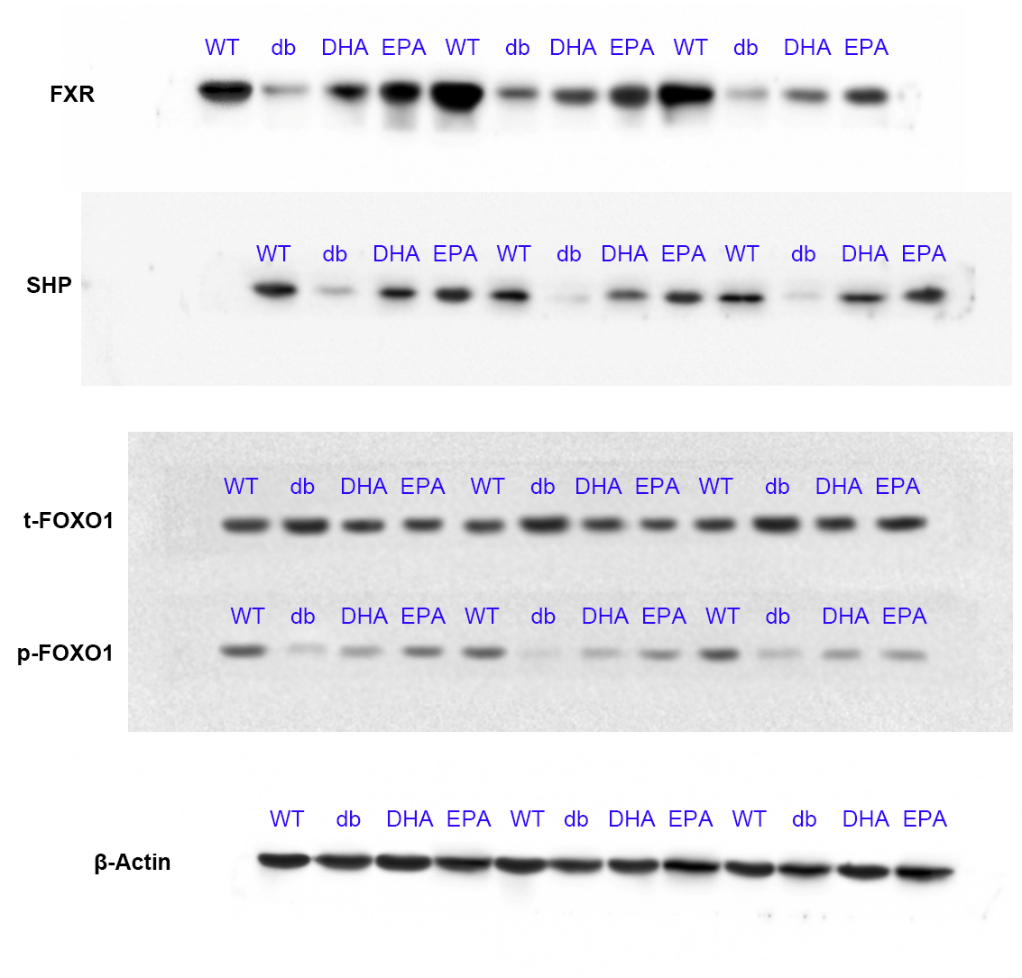
**

**
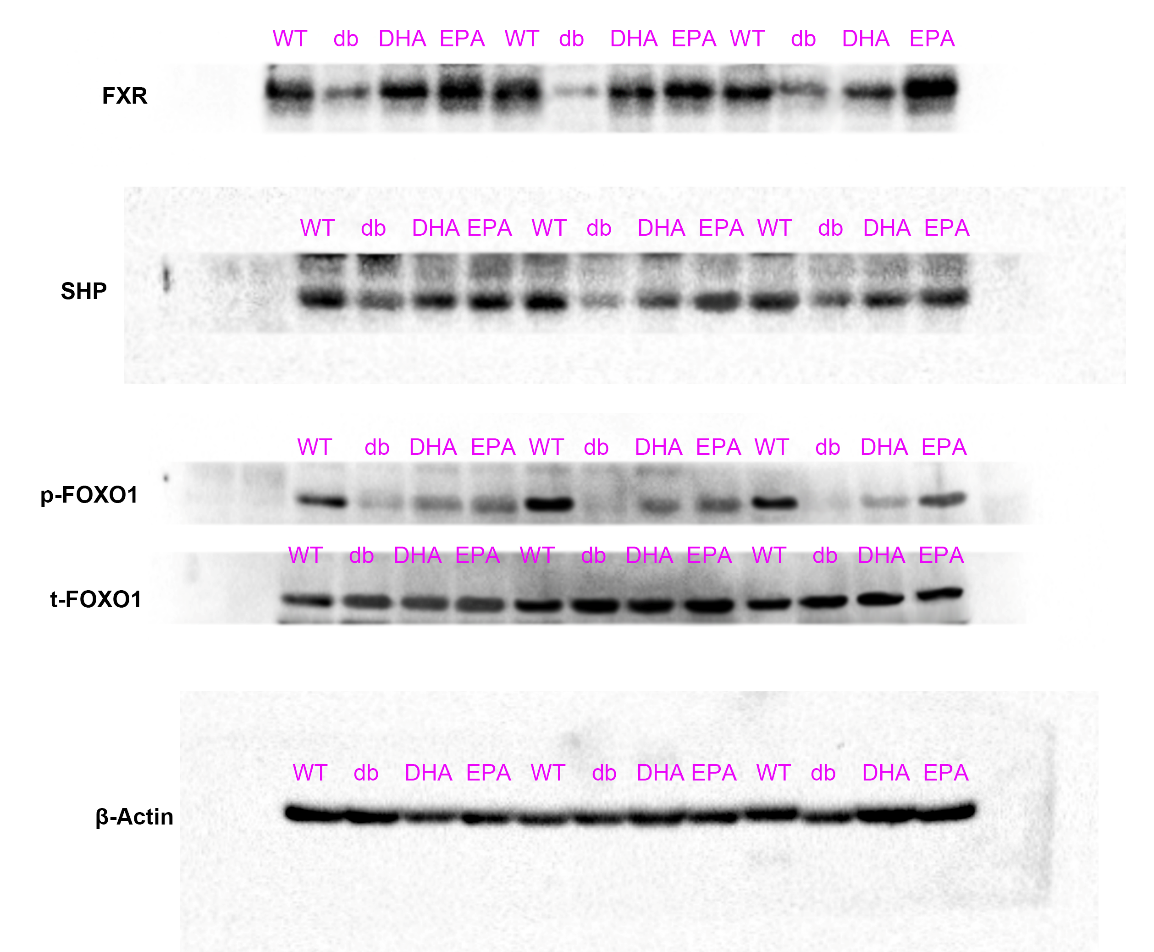
**

Figure S13. Unedited immunoblots for Fig. 5g. Immunoblots are labeled in blue for male and pink for female (*n*=3 male and *n*=3 female per group).

**
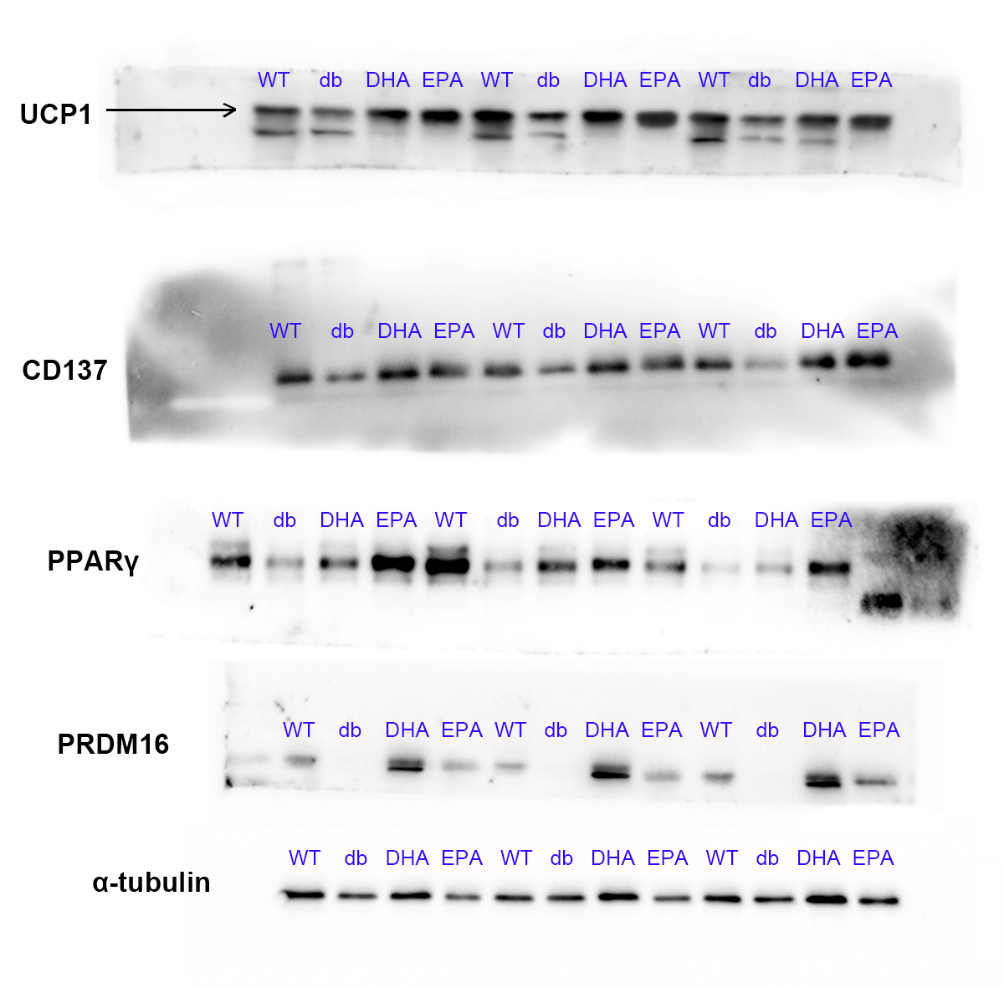
**

**
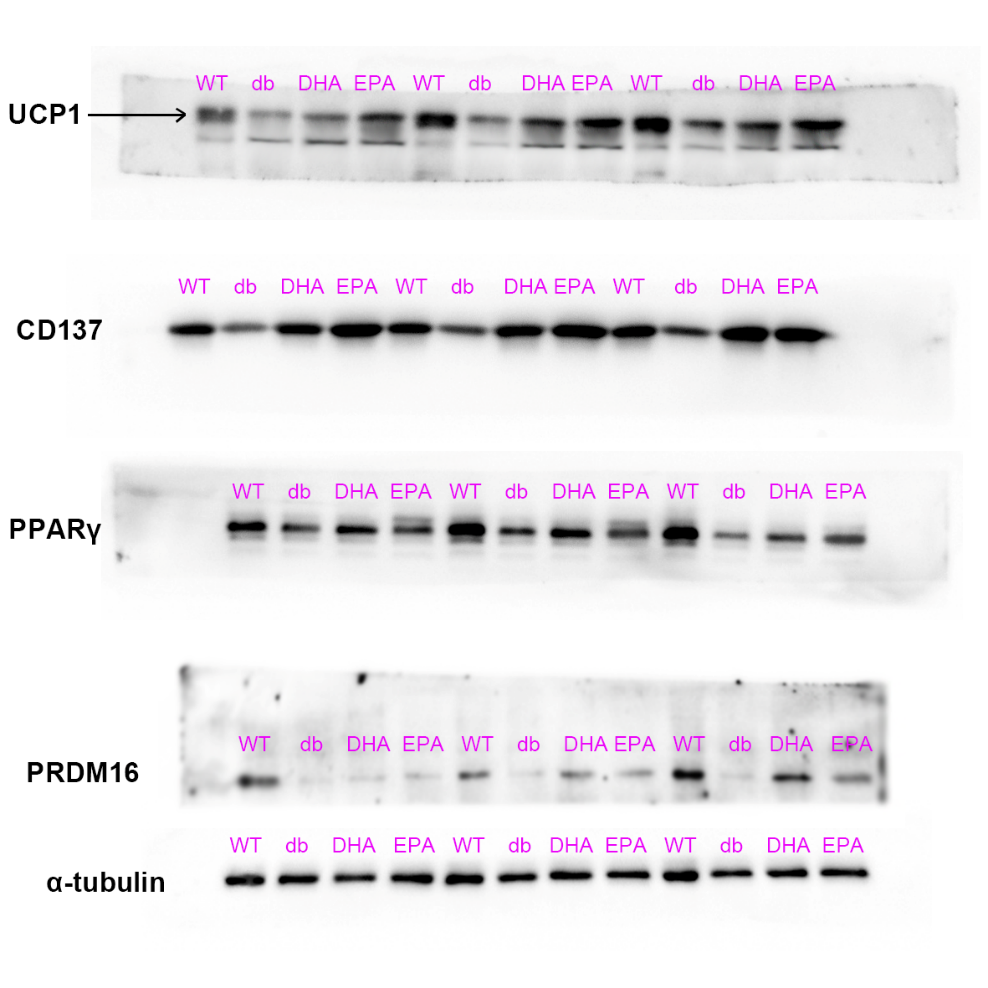
**

Figure S14. Unedited immunoblots for Fig. 6g. Immunoblots are labeled in blue for male and pink for female (*n*=3 male and *n*=3 female per group).

**
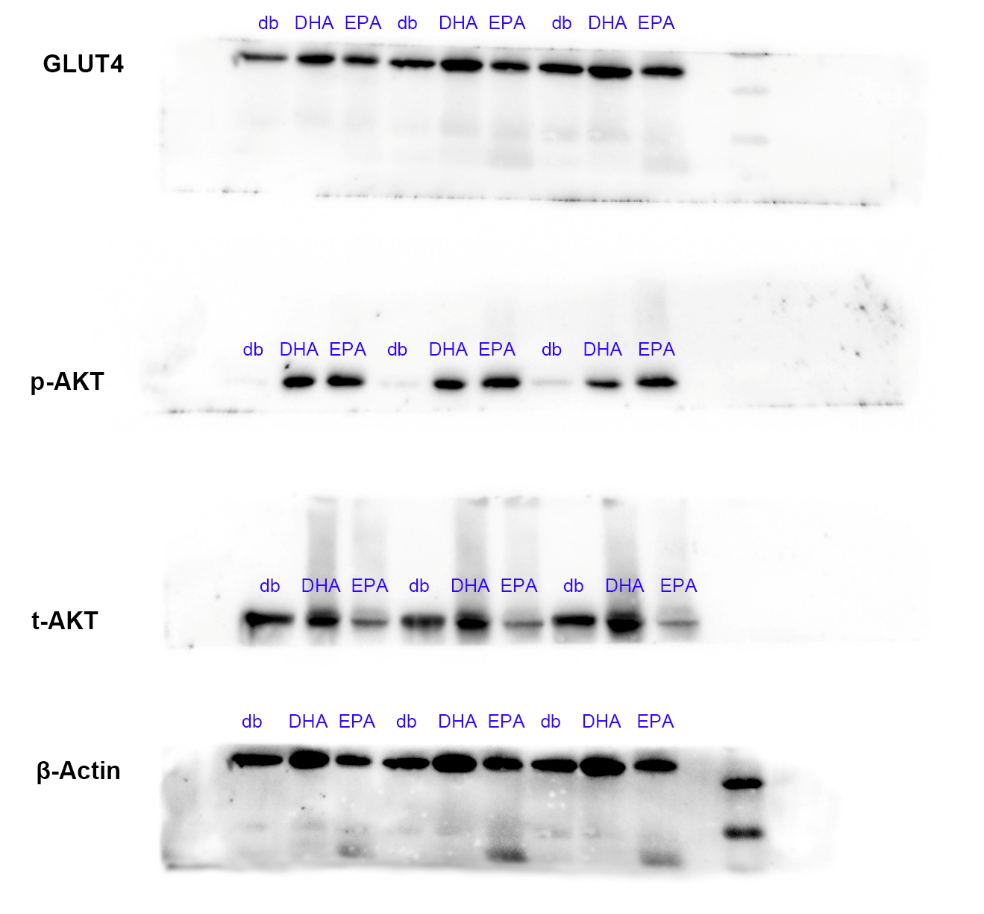
**

**
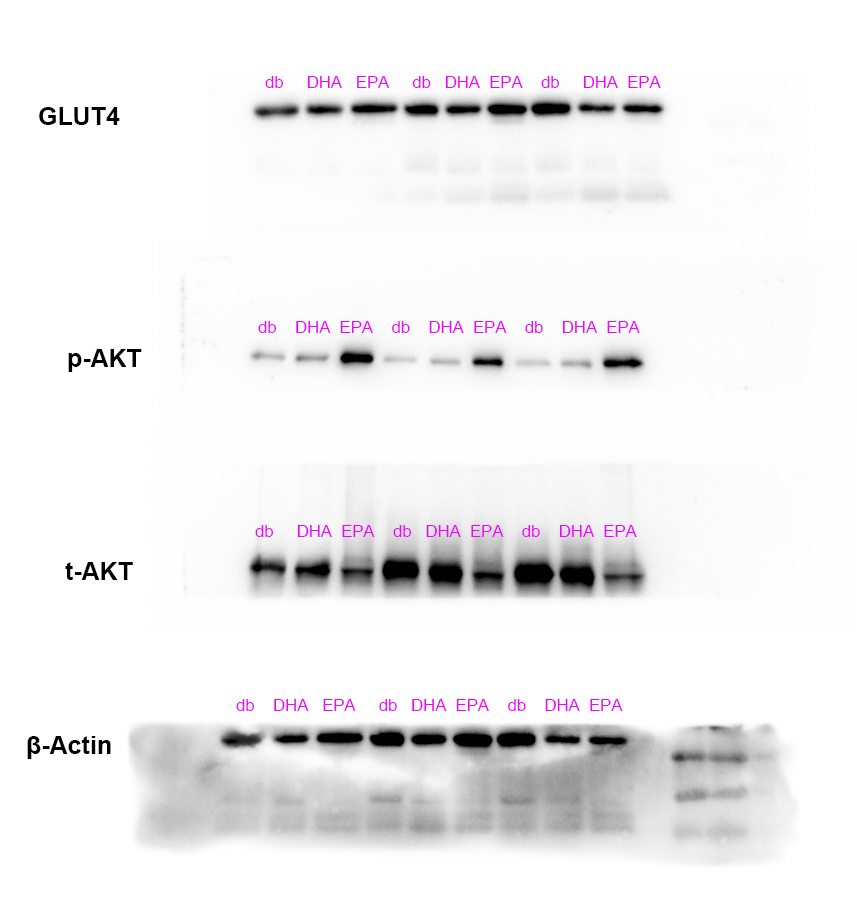
**

Figure S15. Unedited immunoblots for Fig. 6h. Immunoblots are labeled in blue for male and pink for female (*n*=3 male and *n*=3 female per group).
