# Supplementary material for: Eicosapentaenoic and docosahexaenoic acids attenuate hyperglycemia through the microbiome-gut-organs axis in db/db mice
Source: Microbiome. 2021 Sep 10;9:185. doi: 10.1186/s40168-021-01126-6 (PMC8434703; doi:10.1186/s40168-021-01126-6)
Supplement: Supplementary file 3 — Additional file 2: Supplementary methods [file 40168_2021_1126_MOESM3_ESM.docx]

# Additional file 2

**Supplementary methods.**

**Animals and diets**

Animal experiments were approved by the Zhejiang Chinese Medical University Animal Care and Use Committee (approval no. ZSLL-2018-016). Four-week-old C57BL/KsJ-*db/db* mice and C57BL/6 mice were purchased from the Model Animal Research Center of Nanjing University (Nanjing, Jiangsu, China) and were individually housed in ventilated cages in the animal facility of Zhejiang Chinese Medical University (Hangzhou, China) with a 12 h light-dark cycle, free access to food and water, and standard conditions for temperature (23-25 °C) and humidity (50–60%). After 7 days of acclimation, the *db/db* mice were randomly assigned to one of three diet groups (12 mice for each group): the normal control diet (AIN93G; Research Diets, Inc., USA) (*db/db*); control diet supplemented with 1% (w/w) of DHA (purity >99%; Larodan Fine Chemicals, Malmo, Sweden) (*db/db*+DHA); and control diet supplemented with 1% (w/w) of EPA (purity >99%; Larodan Fine Chemicals) (*db/db*+EPA) for 10 weeks. A group of C57BL/6J mice (WT) was also fed a control diet for 10 weeks as the positive control. The compositions of diets are shown in **Additional file 1: Table S1**. Body weight and food intake were measured weekly by an electrical balance. After the treatment, mice were euthanized and blood samples were collected. Tissues were carefully dissected and flash frozen in liquid nitrogen.

For the experiment of short-chain fatty acids administration, eighteen male C57BL/KsJ-*db/db* mice (6-week-old) were randomly assigned to three groups (n=6 per group). The *db/db*+propionate group was gavaged with 80 mg sodium propionate per day and the *db/db*+ butyrate group was gavaged with 80 mg sodium butyrate per day [1] for 4 weeks, while the *db/db* group received vehicle (saline). Mice in all groups were kept continually on the normal control diet (AIN93G; Research Diets, Inc., USA) for the whole experiment. After the treatment, mice were euthanized and samples of intestinal tissue were collected.

**Indirect calorimetry**

At the 9^th^ week of treatment, metabolic parameters including oxygen consumption, carbon dioxide consumption, and respiratory exchange ratio (RER) were measured by an 8-cage animal monitoring system (PhenoMaster/LabMaster, TSE Systems, Bad Homburg, Germany) at room temperature under 12-h day/night cycles. Mice were acclimated in the metabolic cages for 2 days prior to data collection.

**Glucose, insulin, and pyruvate tolerance tests**

Oral glucose tolerance test (OGTT), insulin tolerance test (ITT) and pyruvate tolerance test (PTT) were performed during the last week of the intervention. After overnight fasting, mice were orally gavaged with 66% glucose solution (OGTT, 3 g/kg BW) or injected i.p. with sodium pyruvate (PTT, 2 g/kg BW). For ITT, mice were injected i.p. with insulin (0.75 U/kg BW,) after fasting for 6 hours. After glucose gavage, pyruvate injection, or insulin injection, blood glucose levels in the blood samples from tail veins were measured immediately before (*t* = 0 min) and at selected time points using an Accu-Check glucose meter (Roche).

**Biochemical analyses**

The serum glucose, TG, TC, high-density lipoprotein cholesterol, and low-density lipoprotein cholesterol were measured using a 7020 automatic biochemistry analyzer (Hitachi Ltd., Tokyo, Japan). The whole blood Glycosylated hemoglobin (HbA1c) concentration was determined by the ionic exchange high-performance liquid chromatography method [2]. The serum insulin, LPS, TNF-α and IL-6, intestine GLP-1, and hepatic PEPCK, G6PC and GP levels were detected using commercial enzyme linked immunosorbent assay kits (Cusabio Biotech Co. Ltd., Wuhan, Hubei, China) according to manufacturer’s protocol. Liver TG was isolated and quantified using a colorimetric assay kit (Applygen, Beijing, China) according to the manufacturer's instructions. Hepatic cholesterols including TC, HDL-C, and LDL-C were assessed using commercial ELISA kits (Cusabio Biotech).

**Fatty acid analysis**

Analysis of the marine n-3 fatty acid content in adipose tissue was performed as described previously [3]. Lipids were extracted from gonadal adipose tissues using a chloroform/methanol mixture (2:1, v/v) followed by saponification and methylation. The fatty acid methyl esters were separated on the Agilent 7890A GC with a flame ionization detector and a capillary column (DB-23, Agilent Technologies, USA) and were identified by authentic standards (Nu-Chek Prep, USA).

**SCFA analysis**

SCFA were quantified as previously published [4]. Briefly, the cecal content was diluted 1:5 (v/v) with ultrapure water and homogenized by vortexing. After centrifugation (10000 ×g, 4 °C) for 10 min, the supernatant was deproteinized with 1% sulphosalicylic acid and diluted with ultrapure water. Subsequently, the resultant samples were filtered and then transferred into autosampler vials before being analyzed by the Agilent 7890A GC with the DB-FFAP column (30 m × 0.25 mm i.d., 0.25 μm, Agilent Technologies). SCFA concentrations were quantified using the calibration curves prepared with different concentrations of acetate, propionate, butyrate, and valeric acid (Sigma-Aldrich, St. Louis, MO).

**Morphometric analysis, immunohistochemistry and immunofluorescence**

Freshly isolated tissues were fixed in 4% formaldehyde and then embedded in paraffin using a tissue-embedding procedure. Portions of liver tissue sections (3-5 μm) were stained with haematoxylin and eosin (H-E), pancreas tissue sections were stained with anti-insulin (1:64000; ab181547, Abcam), and adipose tissue sections were stained with UCP1 (1:5000; ab209483, Abcam) or CD137(1:1000; ab203391, Abcam) following standard protocols. Liver sections were also incubated with anti-F4/80 (1:100; ab100790, Abcam) overnight at 4°C for macrophage identification. Images were captured on an N-SIM super-resolution microscope system (Nikon Co., Tokyo, Japan) and analyzed using the NIS-Elements C software.

**Apoptosis assay**

Islets of mice were isolated and the apoptosis of β-cells was quantified with the Cell Death Detection ELISA assay (Roche Applied Science) according to manufacturer’s protocols. Data were expressed as fold changes over the control groups.

**Gene expression**

Total RNA was extracted from the small intestine, WAT, liver and pancreas using TRIzol (Invitrogen, San Diego, CA). Subsequently, equal amounts of total RNA were reverse-transcribed into cDNA with the RT reagent kit (Takara Bio Inc., Shiga, Japan). The mRNA expression was quantified by a two-step qRT-PCR method [5] using SYBR Green on the CFX-96 RT-PCR detection system (Bio-Rad Laboratories). Sequences of the used primers are provided in **Additional file 1: Table S2**. Expression was normalized to the housekeeping gene glyceraldehyde-3-phosphate dehydrogenase.

**Western blot**

Total cellular protein from liver and WAT was extracted by lysing frozen tissues in RIPA lysis buffer (Millipore, Boston, MA) containing inhibitors of protease and phosphatase (Fdbio Science, Hangzhou, China). Total proteins were denatured, resolved by SDS-PAGE and then transferred to PVDF membranes (Millipore). The membranes were blocked using Tris-buffered saline Tween containing 5% bovine serum albumin at room temperature for 1 h and subsequently incubated with the primary antibodies against FXR (1:1000; ab235094, Abcam), FOXO1 (1:1000; ab52857, Abcam), p-FOXO1 (1:500; ab131339, Abcam), SHP (1:1000; ab186874, Abcam), UCP1 (1:5000; ab209483, Abcam), CD137(1:1000; ab203391, Abcam), PRDM16 (1:1000; ab106410, Abcam), PPARγ (1:1000; ab59256, Abcam), GLUT4 (1:200; ab35826, Abcam), p-Akt^Ser473^ (1:1000; #4060L, Cell Signaling) or Akt (1:1000; 4691L, Cell Signaling) overnight at 4 °C. The loading control was β-Actin (1:1000; ab8226, Abcam) or α-tubulin (1:1000, E021030, EarthOx Life Sciences). Images were captured using the ChemiScope 3300 Mini Chemiluminescence Imaging System (Clinx Science Instruments Co., Ltd., Shanghai, China).

**Fecal microbiota transplantation**

Fecal transplantation was performed in the last 4 weeks of the dietary intervention based on an established protocol [6]. During the 4 weeks, stools from the donors in different groups (*db/db*, *db/db*+DHA and *db/db*+EPA) were collected daily under a laminar flow hood in sterile conditions. Every 100 mg fresh stools were resuspended in 1 ml of sterile saline and intensively mixed by vortexing. Subsequently, the mixture was centrifugated at 800 g for 3 min and the supernatant was used as the transplant material which was prepared on the same day of transplantation within 10 min before gavage. Recipient mice were 4-week-old *db/db* mice fed with control diet for 6 weeks synchronized with the *db/db* group. Before fecal transplantation, their endogenous microbiota were depleted by oral gavage with 200 μl mixed antibiotic solution (ampicillin, 1 g/l; metronidazole, 1 g/l; vancomycin, 0.5 g/l; neomycin, 0.5 g/l) daily for 3 days. The bacterial loads and microbial composition of fecal after the antibiotic treatment in *db/db* mice were also evaluated to validate the process for further fecal microbiota transplantation. During the last 8 h of antibiotic gavage, mice were fed with either control diet or DHA/EPA-enriched diet to facilitate subsequent colonization. The recipient mice were fed with control diet and treated with 200 μl transplant material daily by oral gavage for 4 weeks.

**Gut microbiome analysis**

Gut microbiome analysis was performed using high-throughput 16S rDNA sequencing as described previously [7]. Briefly, DNA was extracted from fecal samples using the QIAamp Fast DNA Stool Mini Kit (Qiagen, Valencia, CA, United States) according to the manufacturer’s handbook. DNA concentration and integrity were determined and the V3-V4 region of the bacterial 16S rRNA was amplified by PCR to construct an amplicon sequencing library. The amplicon was standardized and purified using Agencourt AMPure XP beads (Beckman Coulter, Inc., Brea, CA, United States) before being paired-end sequenced by an Illumina MiSeq platform according to manufacturer’s protocols. Raw sequencing data were subjected to filtration using Trimmomatic, FLASH, and QIIME software. Sequences were assigned to operational taxonomic units (OTUs) at 97% similarity using UPARSE software. Representative sequences were chosen for each OTU with the QIIME package, and taxonomic data were then assigned to each representative sequence using Ribosomal Database Project (RDP) Classifier v.2.2, trained on the Silva database version 123. Those occurring at a relative abundance ≥ 0.002% of total reads in at least one sample were further analyzed. The OTU table was rarified and Chao 1 metric was calculated by QIIME to estimate α-diversity. Subsequently, the microbial community clustering (β-diversity) was estimated by principal coordinate analysis (PCoA) using weighted and unweighted UniFrac distance matrices. The linear discriminant analysis (LDA) effect size (LEfSe) analysis (LDA > 2) was used to identify differential taxa of biological relevance between groups. Non-parametric analysis was performed to assess significant differences in specific taxa between groups using the Kruskall-Wallis test followed by Mann-Whitney test.

**Microbial functional profiles prediction**

The microbial functional profiles of DHA/EPA-altered gut microbiota were predicted using PICRUSt software (<http://picrust.github.io/picrust>). This method predicts the gene family abundance from the phylogenetic information with an estimated accuracy of 0.8. The OTU BIOM table of microbiome was used as an input file for metagenome imputation and was first rarefied to an even sequencing depth prior to the PICRUSt analysis. The resulting OTU table was normalized by 16S rRNA gene copy number. Then, the gene content was predicted for each individual and the KEGG Orthology (KO) profile was derived by summing up the relative abundance of genes which aligned to the same KO. Predicted gene class abundances were analyzed at KEGG Orthology group levels 3[8]. Results from PICRUSt were further analyzed using Statistical Analysis of Metagenomic Profiles (STAMP).

**Metabolomic profiling analysis**

**Extraction of metabolites.** The sample preparation methods were described previously [9]. Briefly, fecal samples were soaked in methanol (1/3, M/V) for 24 h, followed by ultrasound, centrifugation and a step filtration (10 kDa). Serum sample aliquots were treated with methanol (1/4, V/V) and vortexed for 1min. The mixture was kept at −20 ℃ for 30 min followed by centrifugation (4,000 g for 20 min) and filtration.

**UHPLC-Q-Orbitrap-HRMS.** UHPLC-Q-Orbitrap-HRMS analyses were performed using a quadrupole-orbitrap mass spectrometer (Q-Exactive, Thermo Fisher Scientific, Waltham, MA, USA) equipped with Dionex 3000 Ultimate UHPLC and autosampler (Thermo Fisher Scientific). Each injected solution (5 μL) was separated with the Acquity UPLC HSS T3 column (2.1×150 mm i.d., 1.8 µm) together with an HSS T3 VanGuard Pre-column (2.1×5 mm i.d., 1.8 µm) at 35 °C. Gradient elution was performed with a mixture of 0.1% formic acid in water (mobile phase A) and 0.1% formic acid in acetonitrile (mobile phase B) at a flow rate of 0.3 mL/min. The UHPLC analysis followed a gradient elution program. In detail, the ingredient percentage of solvent A was initially held at 98% for 3 min, then followed by a linear gradient from 98 to 0% over a 12-min period and maintained for 3 min, then returned to 98% within 0.5 min and held for 2.5 min. High resolution mass spectrometry analyses were performed with a heat electrospray ionization (HESI) source under both positive ion mode and negative ion mode. Ionization conditions were set as follows: sheath gas flow rate, 40 Units; auxiliary gas flow rate, 10 Units; spray voltage, 3.5 kV in positive ion mode and 3.4 kV in negative ion mode; capillary temperature, 320 °C; s-lens RF level, 50; and auxiliary gas heater temperature, 350 °C. The acquisition mode of quadrupole-orbitrap analyses was set to be the full MS/dd-MS2 (TopN) mode, which is a combination of full MS mode and dd-MS2 mode. Full MS mode employed a mass scan range of 60-900 m/z, an orbitrap resolution of 70,000 with maximum latency time of 100 ms, and automatic gain control (AGC) target of 3×106. The dd-MS2 mode employed an orbitrap resolution of 17,500 with maximum latency time of 50 ms, AGC target of 1×105, loop count of 5, high energy collisional dissociation stepped normalized collision energy of 20, 40 and 60, and isolation window of 1.5 m/z. Data analyses were performed using the Xcalibur 4.1 and Compound Discoverer 2.1 software (Thermo Fisher Scientific).

**Data processing**. The Compound Discovery v2.1 software (Thermo Fisher Scientific) was used to process and analyze the Xcalibur ‘.raw’ data files generated from the UHPLC-Q-Orbitrap-HRMS. To start the data processing task, a new project was created and the non-targeted metabolomics workflow was selected. Then, the ‘.raw’ data was added into the project. The quality control (QC) samples were prepared by pooling of each extracted fecal or serum samples and were measured when every 6 samples were tested. Given that the measurement errors of a single batch are randomly distributed, the mean or median levels of a batch of QC samples can be used to compare and correct the measurement errors of different batches. The Compound Discovery software was used to process the batch normalization automatically using the data from QC samples. The workflow parameters were set as follows: mass tolerance, 5ppm; S/N threshold, 3; retention time (RT) tolerance, 0.2min; minimal peak intensity, 2×105; maximal RSD of QC area, 30%.

**Identification of metabolites**. Based on the data from mzCloud (https://www.mzcloud.org) and Human Metabolome Database (HMDB) (http://www.hmdb.ca), the structural formula of metabolites were identified according to the accurate mass of their MS and tandem MS spectra. Part of metabolites was verified by standards available in our lab.

**Data analysis.** After normalization (by weight of fecal or serum samples), all the extracted UHPLC-Q-Orbitrap-HRMS ions (both positive and negative ion modes) were imported into SIMCA software (version 14.1, Umetrics, Umea, Sweden) for statistical analysis. Principle component analysis (PCA) and partial least squares discriminant analysis (OPLS-DA) with unit variance (UV) scaling were carried out to discriminate DHA/EPA treated mice from control mice. Fold changes were calculated as mass response ratio between two arbitrary groups (*db/db*+DHA vs. *db/db* or *db/db*+EPA vs. *db/db*). The variable contribution of the OPLS-DA model was ranked by the variable importance in the projection (VIP). Metabolites passing the threshold of VIP > 1 were considered as significantly different between the DHA/EPA-fed and control mice groups, which was validated at a univariate level with *P* < 0.05 and fold change ≥1.2 or ≤0.8. All the annotated differential metabolites were uploaded to MetaboAnalyst (https://www.metaboanalyst.ca/) to identify perturbed pathways according to Kyoto Encyclopedia of Genes and Genomes (KEGG) pathway database (https://www.genome.jp/kegg/). Based on the application of MetaboAnalyst 4.0 and ‘Rattus norvegicus’ library, we used the hypergeometric test for over representation analysis and the relative-betweenness centrality for pathway topology analysis to investigate individual pathway enrichment as suggested by the profile information of DHA/EPA associated metabolites.

**Bacterial culture *in vitro***

Strains of *Escherichia coli* MG1655 and *Coriobacterium glomerans* ATCC 49209 were used. *Escherichia coli* was grown aerobically in LB medium (Meilunbio, Dalian, China) and *Coriobacterium glomerans* was grown anaerobically in Gifu anaerobic medium (GAM) [10] (Coolaber, Beijing, China) at 37 °C. At mid-exponential phase of bacterial growth (measured by spectrophotometry), cultures were washed and diluted into fresh media at a final concentration of 10^8^-10^9^ CFUs mL^−1^. Then, the growth media were added with DHA (200 µM), EPA (200 µM) or vehicle (EtOH) and incubated at 37 °C for 8-72 h. The cell density of the cultures was monitored by recording OD_600_ values [11] of samples taken at various time points in order to determine the bacterial growth until the stationary growth phase.

**Statistical analysis**

All data were presented as mean ± SEM. Differences between two groups were analyzed by two-tailed Student’s *t*-test. Differences between three or more groups were evaluated through one-way analysis of variance (ANOVA) followed by Tukey’s multiple comparison post-test. Two-way ANOVA was used for data from indirect calorimetry experiments. Heat maps were generated using STAMP. The Spearman’s rho nonparametric correlations between specific taxa and fecal metabolites were calculated using the SAS statistical package (version 9.4, SAS Institute). Gephi Graph Visualization and Manipulation software version 0.9.2 was used to visualize the network. *P*<0.05 was considered statistically significant.

**References**

1. Jia Y, Hong J, Li H, Hu Y, Jia L, Cai D, et al. Butyrate stimulates adipose lipolysis and mitochondrial oxidative phosphorylation through histone hyperacetylation-associated β3-adrenergic receptor activation in high-fat diet-induced obese mice. Exp Physiol. 2017;102(2):273-81.

2. Kondo N, Shibayama Y, Toyomaki Y, Yamamoto M, Ohara H, Nakano K, et al. Simple method for determination of A1c-type glycated hemoglobin(s) in rats using high performance liquid chromatography. J Pharmacol Methods. 1989;21(3):211-21.

3. Zhu J, Zhuang P, Luan L, Sun Q, Cao F. Preparation and characterization of novel nanocarriers containing krill oil for food application. J Funct Foods. 2015;19:902-12.

4. Zhuang P, Shou Q, Lu Y, Wang G, Qiu J, Wang J, et al. Arachidonic acid sex-dependently affects obesity through linking gut microbiota-driven inflammation to hypothalamus-adipose-liver axis. Biochim Biophys Acta Mol Basis Dis. 2017;1863(11):2715-26.

5. Bustin SA, Benes V, Garson JA, Hellemans J, Huggett J, Kubista M, et al. The MIQE guidelines: minimum information for publication of quantitative real-time PCR experiments. Clin Chem. 2009;55(4):611-22.

6. Chang C-J, Lin C-S, Lu C-C, Martel J, Ko Y-F, Ojcius DM, et al. Ganoderma lucidum reduces obesity in mice by modulating the composition of the gut microbiota. Nat Commun. 2015;6:7489.

7. Ye J, Lv L, Wu W, Li Y, Shi D, Fang D, et al. Butyrate protects mice against methionine-choline-deficient diet-induced non-alcoholic steatohepatitis by improving gut barrier function, attenuating inflammation and reducing endotoxin levels. Front Microbiol. 2018;9:1967.

8. Langille MGI, Zaneveld J, Caporaso JG, McDonald D, Knights D, Reyes JA, et al. Predictive functional profiling of microbial communities using 16S rRNA marker gene sequences. Nat Biotechnol. 2013;31(9):814-21.

9. Cesbron N, Royer AL, Guitton Y, Sydor A, Le Bizec B, Dervilly-Pinel G. Optimization of fecal sample preparation for untargeted LC-HRMS based metabolomics. Metabolomics. 2017;13(8):99.

10. Wu J, Liu M, Zhou M, Wu L, Yang H, Huang L, et al. Isolation and genomic characterization of five novel strains of Erysipelotrichaceae from commercial pigs. BMC Microbiol. 2021;21(1):125.

11. Miajlovic H, Fallon PG, Irvine AD, Foster TJ. Effect of filaggrin breakdown products on growth of and protein expression by Staphylococcus aureus. J Allergy Clin Immunol. 2010;126(6):1184-90.e3.
